# Formal Modelling of Toll like Receptor 4 and JAK/STAT Signalling Pathways: Insight into the Roles of SOCS-1, Interferon-β and Proinflammatory Cytokines in Sepsis

**DOI:** 10.1371/journal.pone.0108466

**Published:** 2014-09-25

**Authors:** Rehan Zafar Paracha, Jamil Ahmad, Amjad Ali, Riaz Hussain, Umar Niazi, Samar Hayat Khan Tareen, Babar Aslam

**Affiliations:** 1 Atta-Ur-Rahman School of Applied Biosciences (ASAB), National University of Sciences and Technology (NUST), Islamabad, Pakistan; 2 Research Center for Modeling and Simulation (RCMS), National University of Sciences and Technology (NUST), Islamabad, Pakistan; 3 Shifa College of Pharmaceutical Sciences, Shifa Tameer-e-Millat University, Islamabad, Pakistan; 4 IBERS, Aberystwyth University, Edward Llwyd Building, Penglais Campus, Aberystwyth, Ceredigion, Wales, United Kingdom; University of Catania, Italy

## Abstract

Sepsis is one of the major causes of human morbidity and results in a considerable number of deaths each year. Lipopolysaccharide-induced sepsis has been associated with TLR4 signalling pathway which in collaboration with the JAK/STAT signalling regulate endotoxemia and inflammation. However, during sepsis our immune system cannot maintain a balance of cytokine levels and results in multiple organ damage and eventual death. Different opinions have been made in previous studies about the expression patterns and the role of proinflammatory cytokines in sepsis that attracted our attention towards qualitative properties of TLR4 and JAK/STAT signalling pathways using computer-aided studies. René Thomas’ formalism was used to model septic and non-septic dynamics of TLR4 and JAK/STAT signalling. Comparisons among dynamics were made by intervening or removing the specific interactions among entities. Among our predictions, recurrent induction of proinflammatory cytokines with subsequent downregulation was found as the basic characteristic of septic model. This characteristic was found in agreement with previous experimental studies, which implicate that inflammation is followed by immunomodulation in septic patients. Moreover, intervention in downregulation of proinflammatory cytokines by SOCS-1 was found desirable to boost the immune responses. On the other hand, interventions either in TLR4 or transcriptional elements such as NFκB and STAT were found effective in the downregulation of immune responses. Whereas, IFN-β and SOCS-1 mediated downregulation at different levels of signalling were found to be associated with variations in the levels of proinflammatory cytokines. However, these predictions need to be further validated using wet laboratory experimental studies to further explore the roles of inhibitors such as SOCS-1 and IFN-β, which may alter the levels of proinflammatory cytokines at different stages of sepsis.

## Introduction

Sepsis is a serious medical condition associated with complications of an exacerbated human immune response against endotoxin/lipopolysaccharides (LPS) mediated severe infections [1]. It can lead to endotoxin shock, organ damage, morbidity and eventual death [2], [3]. The incidence of sepsis is growing regardless of advances in the therapeutic and supportive treatments [4], [5]. In 1992, nearly 500,000 cases of sepsis were found in the United States among which 35% of the patients led to mortality [6]. In 2001, around 750,000 cases of sepsis with 28.6% mortality rate per annum was recorded [7]. In a trend analysis from 1993 to 2003, a significant increase in the cases of severe sepsis and hospitalization was reported [8], which is still rising [9].

Pro- and anti-inflammatory cytokines are groups of proteins, which mediate endogenous inflammation and immunomodulation, respectively. Proinflammatory cytokines (PICyts) including tumour necrosis factor (TNF)-α, interferon (IFN)-γ, interleukin (IL)-la, IL-1β and IL-6 induce a series of immune responses to overcome the pathogen load [10]. In contrast, anti-inflammatory cytokines such as transforming growth factor (TGF)-β, IL-4, IL-10, IL-13 and other cytokine inhibitors including soluble tumour necrosis factor receptor (sTNFR)-I and II, IL-lra, or soluble IL-1 receptors (sIL-1r) modulate the immune responses and can induce temporary immunosuppression in septic patients [11].

Our understandings about the contributory role of pro- and anti-inflammatory immune responses in sepsis evolved with the passage of time and highlighted disparities among the scientific findings. Earlier animal studies suggested that proinflammatory responses were the major cause of the systemic inflammatory response syndrome (SIRS) and mortality, whereas anti-inflammatory responses were associated with comparatively less severe complications [12]. In contrast, recently submitted suggestions disprove previous studies and indicated that the anti-inflammatory responses and immunosuppression might in fact be responsible for compensatory anti-inflammatory response syndrome (CARS), severe sepsis, organ damage and subsequent mortality [13]–[17]. Moreover, other studies implicated that pro- and anti-inflammatory responses are correlated with each other and provide opportunities for septic patients for the management of pathogens and hyperinflammation at different levels of sepsis [18], [19].

**Figure 1:**
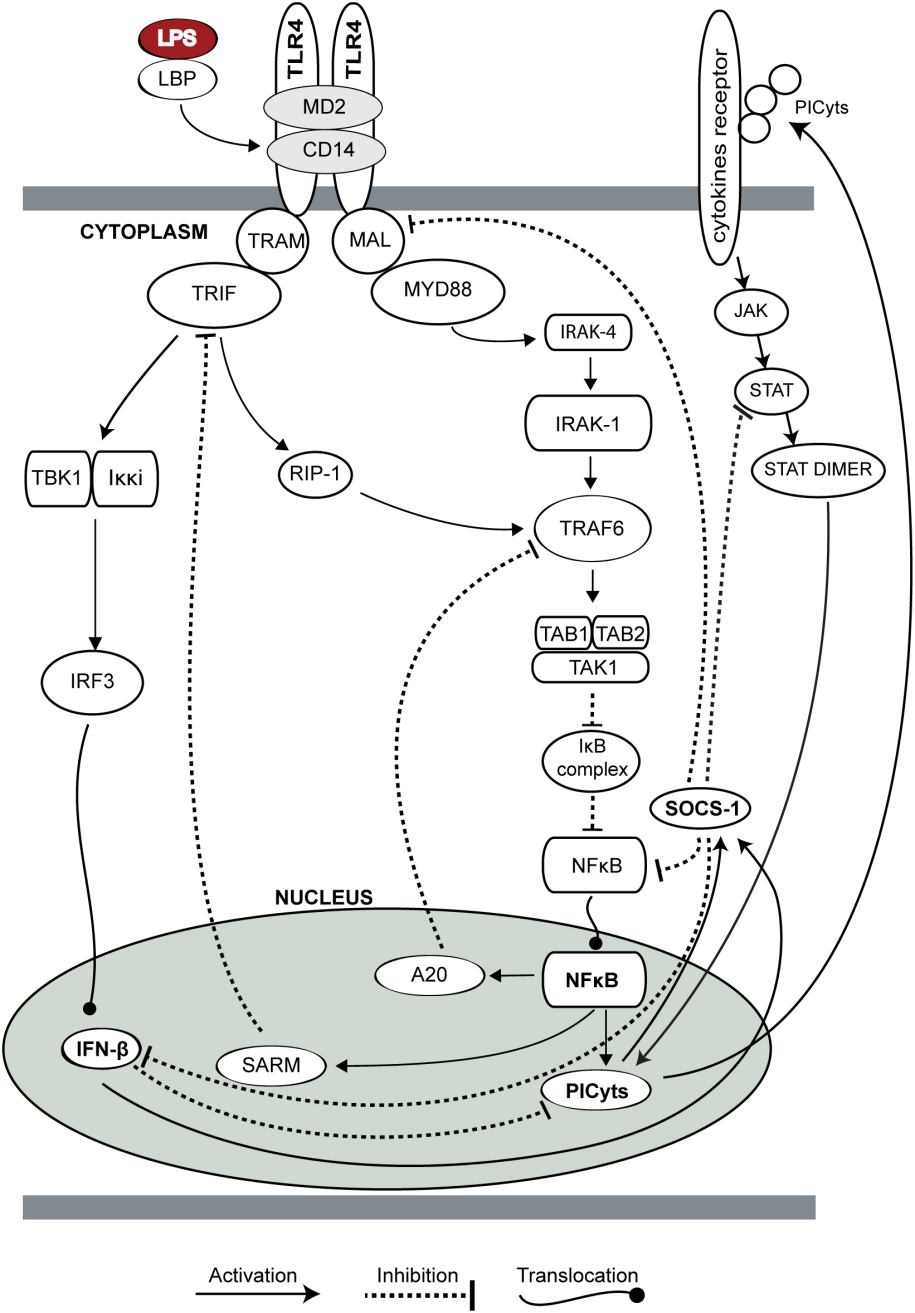
TLR4 and JAK/STAT signalling pathway. Overview of TLR4 and JAK/STAT signalling pathway adopted from previous experimental studies and databases associated with signalling pathways [20], [28]–[33]. TLR4 activates two separate signalling pathways, including MyD88 and TRIF dependent pathways [103]. TIRAP/Mal and TRAM are recruited by TLR4 as adaptor proteins to activate MyD88 and TRIF dependent pathways, respectively [35], [103]. Following MYD88 activation, IRAK4 is phosphorylated by MyD88-MAL complex, which ultimately results in the phosphorylation of IRAK1 protein. Phosphorylated IRAK1 activates TRAF6 [104] which after forming a complex with TAK1-TAB1/2 activates Iκκ complex [105]. Iκκα and Iκκβ catalyse the phosphorylation of IκB, resulting in its dissociation from NFκB. Afterwards NFκB translocate into nucleus [106] and transcribes PICyts which results in the subsequent induction of SOCS-1 [48], [107]. Along with PICyts, SARM and A20 are also transcribed by NFκB which inhibit TRIF and TRAF6, respectively [44], [108]. Interaction of SOCS-1 with MAL results in its polyubiquitylation and degradation of MAL [42]. SOCS-1 also result in the degradation of NFκB after binding with its p65 subunit [109]. Moreover, it is also responsible for inhibiting PICyts mediated JAK/STAT signalling [110]. The alternate pathway for the MyD88 independent induction of NFκB is TRIF which associates with RIP-1 and induce TRAF6 [111]. Cytoplasmic domain of TLR4 associates with TRAM and TRIF, and interacts with a complex of TBK1 and Iκκi to induce phosphorylation of IRF3 [103]. After dimerization, phosphorylated IRF3 translocate into nucleus which results in the production of type I IFNs. IFN-β is responsible for the downregulation of PICyts through a shift of TH1 to TH2 responses and induce immune regulation. Recently SOCS-1 mediated downregulation of IFN-β has been observed [50].

Toll like receptors (TLRs) are pattern recognition receptors and play their important role in the induction of innate immunity against endotoxins [20]. Previous studies have demonstrated that the expression levels of TLR4 were elevated on human monocytes in healthy volunteers challenged with LPS [21] as well as in septic patients [22], [23]. Activation of TLR4 leads to the production of pro- and anti-inflammatory cytokines by inducing two different signalling pathways [24]. TLR4 is unique among other TLRs due to its ability to induce myeloid differentiation primary response gene (MyD)88 and TIR-domain-containing-adapter-inducing interferon-β (TRIF) dependent pathways [25]. These two pathways culminate in the generation of PICyts and Interferon-β (IFN-β), respectively. Along with the production of IFN-β, TRIF dependent signalling has also been implicated to induce the NFκB activation through TRAF6 [26]. On the other hand, IFN-β has been implicated in the modulation of late hyperinflammation in sepsis [27]. Figure 1 is the simple representation of the TLR4 and Janus kinase (JAK)/signal transducer and activator of transcription (STAT) (JAK/STAT) signalling pathways adapted from previous experimental studies and databases associated with biological signalling [28]–[34].

MyD88 dependent pathway activates due to the formation of a complex between MyD88, TLR4 and toll-interleukin 1 receptor (TIR) domain containing adaptor protein (TIRAP also known as MAL). This pathway culminates in the activation of NFκB with subsequent production of PICyts [35]. In contrast, the TRIF dependent pathway is activated by the formation of a complex between TRIF, TRIF related adaptor molecule (TRAM or TICAM2) and TLR4 [36]. This complex results in the activation of transcriptional regulator interferon regulatory factor 3 (IRF3) with subsequent transcription of IFN-β [37]. Moreover, the TRIF dependent pathway has also been implicated for the delayed activation of NFκB, through TRIF mediated TRAF6 activation, however, MyD88 dependent pathway is reported for its explicit contribution in the production of PICyts [38]. PICyts and IFN-β mediated JAK/STAT signalling is essential for the induction of pro- or anti-inflammatory immune responses, respectively [31], [39]. PICyts and IFN-β stimulate JAKs with subsequent translocation of STATs into the nucleus where it transcribes necessary genes responsible to react appropriately against the pathogen or inflammation [30], [31].

TLR4 mediated immune responses are downregulated by several negative feedback mechanisms [40]. Regulation of TLR4 mediated signalling maintain homeostasis between infectious challenge and hyperinflammatory responses [41]. Negative regulatory proteins such as suppressor of cytokine signalling-1 (SOCS-1), A20 zinc finger protein and sterile alpha-and armadillo-motif-containing protein (SARM) are well reported for their inhibitory roles in TLR4 signalling [42]–[44]. Recently, IFN-β is found associated with the induction of TH1 to TH2 response shift to reduce the levels of circulating PICyts [27], [45]–[47]. Various experiments on SOCS-1 knockout cells highlighted that SOCS-1 is necessary to protect against endotoxemia and hyperinflammation by inhibiting PICyts and IFN-β mediated JAK/STAT signalling, respectively [48]–[50].

In this study, we devised qualitative (discrete) model of TLR4 and JAK/STAT signalling, which was constructed by using the well-known mathematical formalism of René Thomas [51]–[53]. Construction of the models according to this formalism do not require quantitative data (the expression of entities and kinetic rate parameters of reactions), which is often difficult to obtain for the biological regulatory networks (BRNs) [54]. Construction of the qualitative model requires only the qualitative thresholds and logical parameters, which can be easily adjusted (see Definitions in Material and Methods section). The qualitative model encompasses all the possible qualitative states or levels of entities present in a BRN. The dynamics of the BRN are captured by the state graph (representing the states and trajectories) where important behaviours can be seen as cyclic paths and paths diverging towards stable states. These cycles and stable states represent the activation profiles of the entities, respectively. The advantage of the state graph is that it can represent a state space in any dimension as a discrete abstraction, while in other approaches, like ordinary differential equations, this may be very challenging. Comparison of the qualitative model with its differential equation counterpart is given by René Thomas et al. in [55] to prove that both approaches are equivalent, however, the qualitative modelling is more suitable for model checking based reasoning to infer unknown parameters [56].

In our previous study, TLR4 mediated MyD88 dependent pathway was studied, with a particular focus on the role of Bruton’s tyrosine kinase and MAL in the production of hyperinflammatory responses especially in the case of cerebral malaria [57]. The current study presents the dynamics of the TLR4 and JAK/STAT signalling pathway by updating our previous study with new interactions and entities to gain an insight into a different pathological condition of sepsis. Additionally, the current study also provides an understanding about the importance of signalling downregulated by SOCS-1, IFN-β, A20 and SARM. Moreover, modelling of interventions in signalling were used to understand the roles of NFκB, PICyts, IFN-β, JAK/STAT and SOCS-1 in immune responses [55].

**Figure 2:**
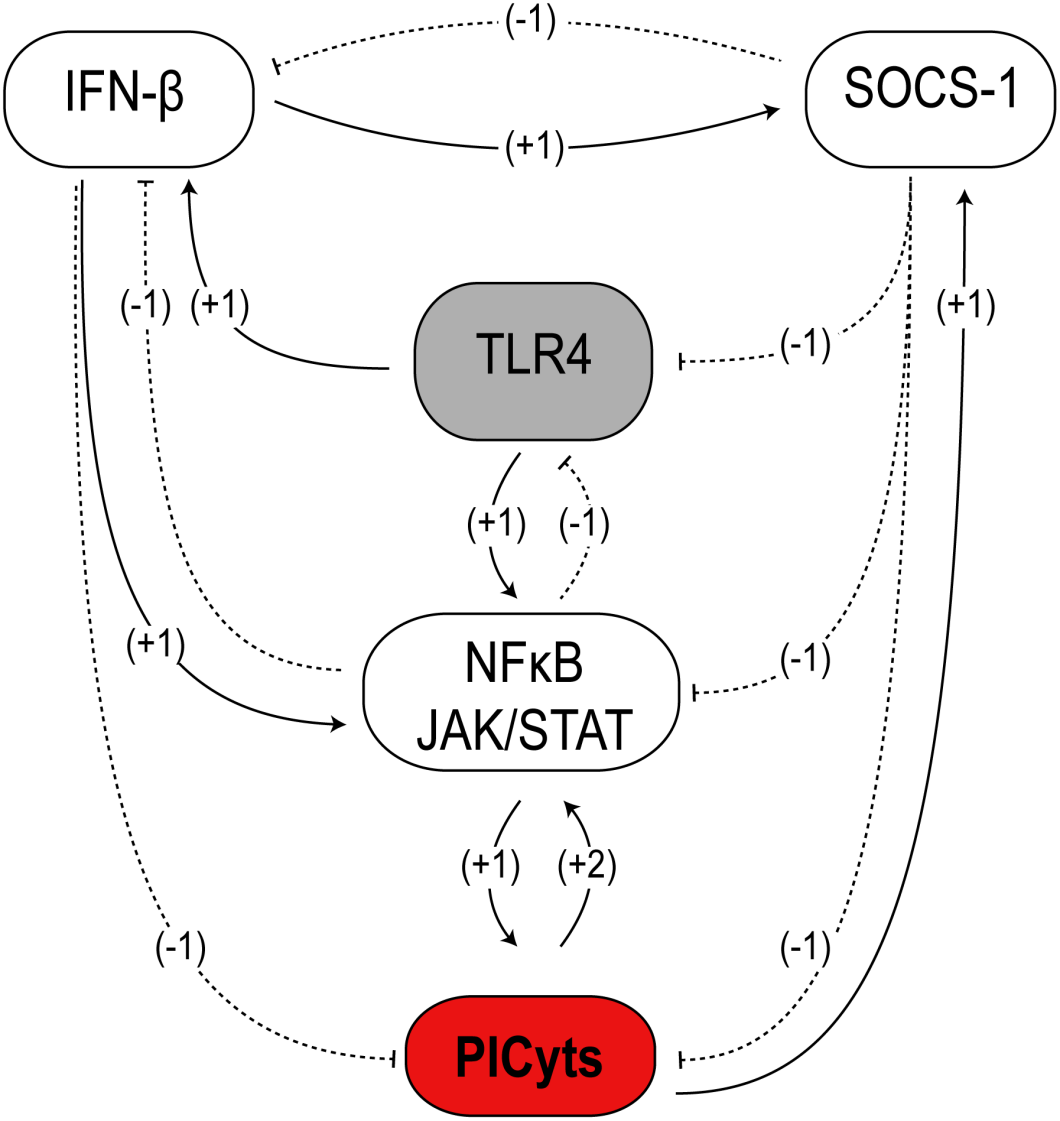
The BRN of TLR4 and JAK/STAT signalling pathway. The reduced BRN of TLR4 and JAK/STAT signalling pathway is derived from Figure 1. Nodes represent entities, whereas interactions between them are shown as edges. Sign on the edges represent the type of interaction between nodes i.e. positive for activation (solid arrows) and negative for inhibition (dotted arrows). Integers “1” and “2” on the edges represent the threshold levels of entities (see Material and Methods section).

The BRN of TLR4-JAK/STAT (Figure 2) in this study implicates that TLR4, IFN-β, JAK/STAT and SOCS-1 mediated signalling perform their roles in a recursive manner. Intervention in the SOCS-1 mediated downregulation of PICyts is associated with the production of overactive immune response, whereas, interventions either in TLR4 or NFκB-JAK/STAT signalling is connected with downregulation of overactive immune responses. Additionally, IFN-β downregulates PICyts in the earlier phase of signalling, whereas SOCS-1 regulates the levels of cytokines in late phase. On this account, levels of PICyts fluctuate within different qualitative levels during sepsis and may provide the basis for the differences in scientific findings [18], [19]. However, these predictions were generated by the use of computer-aided models and need to be further validated in wet laboratory experiments.

## Materials and Methods

### The formalism of René Thomas

Traditionally, biological systems are modelled using ordinary differential equations, which require time derivatives of expression levels, temperature, physical state and kinetic rates of entities, etc. [58]. Due to the complexity of the biological systems, each variable with all of its parameters are either system specific or rarely known. For this reason, computer-aided qualitative modelling of biological systems is generally preferred to understand the dynamics of the BRN at a preliminary level, which can then be validated using *in vitro* experiments.

In 1970, René Thomas introduced Boolean logic for the qualitative modelling of BRNs, which was later generalized to kinetic logic [59]–[66]. Using kinetic logic, possible dynamics of a BRN can be determined in a scalable but rigorous manner. Kinetic logic provided its effectiveness in preference to the Boolean logic by the successful modelling of different BRNs [67]. Effectiveness of the kinetic logic has been proved by analysing lambda phage genetic switch, differentiation process in helper T cells, control of organ differentiation in Arabidopsis thaliana flowers and segmentation during embryogenesis in *Drosophila melanogaster* [51]–[53]. The kinetic logic formalism is an influential method for examining BRNs in which interactions among entities are well reported. Use of logical parameters consistent with threshold values eliminate the necessity of various parameters of expression, temperature, physical state and kinetic rates etc. Moreover, this approach has the ability to model the system close to the approximations obtained by differential equations [55].

### Semantics of the Kinetic Logic Formalism

The semantics of the kinetic logic formalism [55] have been discussed in our previous work [57], where we have explained the following formal definitions by considering an example of a toy BRN composed of three entities (shown as Figure 6 in the previous study). The definitions and the terms necessary to understand the semantics used in this study have been mentioned below, adapted from our previous study [57].

#### Definition 1 (Directed Graph)

“A directed graph is an ordered pair 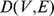, where:

- *V* is the set of all nodes and

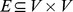
 is the set of ordered pairs called edges or arcs”

An edge e.g. (a,b) is directed from an entity or node “a” to “b”, where “a” is the tail and “b” is the head of that respective edge. In a directed graph, 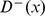 and 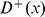 denote the set of predecessors and successors of a specific node 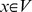, respectively”.

#### Definition 2 (Biological Regulatory Network)

“A BRN is a labelled directed graph 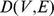, where 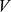 is a set of nodes which represents biological entities and 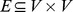 is a set of all possible edges, which represent the interaction between entities”.

- Each edge can be labelled with a pair of variables 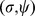, where 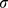 represents the qualitative threshold levels and is a positive integer and 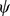 is “+” or “–” representing the type of interaction, which can either be “activation” or “inhibition”, respectively.
- Each node e.g. “a” has a limit (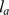), in its threshold level, which is equal to its out-degree (the total number of outgoing edges from “a”). This relation can be presented by 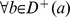 and 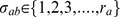 where 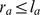 which means that the threshold levels of entity “a” can be set within a range “1” to “total number of outgoing edges” and because it has only one outgoing edge towards predecessor “b” so the threshold level which can be set for it can be only be “1”.
- Each entity, e.g. “a”, has its abstract expression in the set 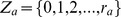.

#### Definition 3 (States)

“The state of a BRN is a tuple 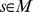, where 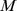 in terms of entity “a” is:
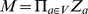



The qualitative states are represented by vector 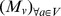, where 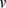 denotes the level of expression of an entity like “a”. According to this definition M is the Cartesian product of the sets of abstract expressions of all entities. A qualitative state represents a configuration of all the elements of a BRN at any instant of time. The number of activators of a particular variable at a given level of expression are represented by its set of resources (see the definition of resources given below)”.

#### Definition 4 (Resources)

“The set of resources 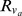 of a variable 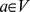 at a level 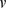 is defined as 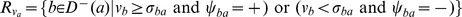. The dynamic behaviours of BRN depends on logical parameters. The set of these logical parameters is defined as 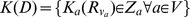.

The parameter 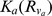 (at a level 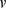 of 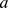) gives the information about the evolution of 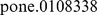. There are three cases: 1) if 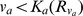 then 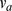 increases by one unit 2) if 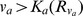 then 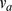 decreases by one unit and 3) if 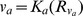 then 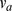 cannot evolve from its current level.

It is convenient to describe the evolution from one level to another by an evolution operator “<$>\raster="rg1"<$>” [68], which is defined in terms of entity “a” as follows:
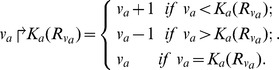



Where 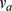 and 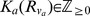
”.

#### Definition 5 (State Graph)

“Let 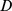 be the BRN and 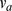 represents the expression level of an entity e.g. “a” in a state 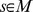. Then the state graph of the BRN will be the directed graph 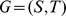, where 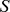 is set of states and 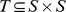 represents a relation between states, called the transition relation, such that 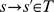 if and only if:

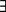
 a unique 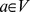 such that 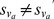 and 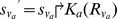 and

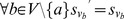
”

According to this definition states evolve asynchronously, thus, in a successor state only one entity changes its level.

### Reduction of the BRN

**Figure 3:**
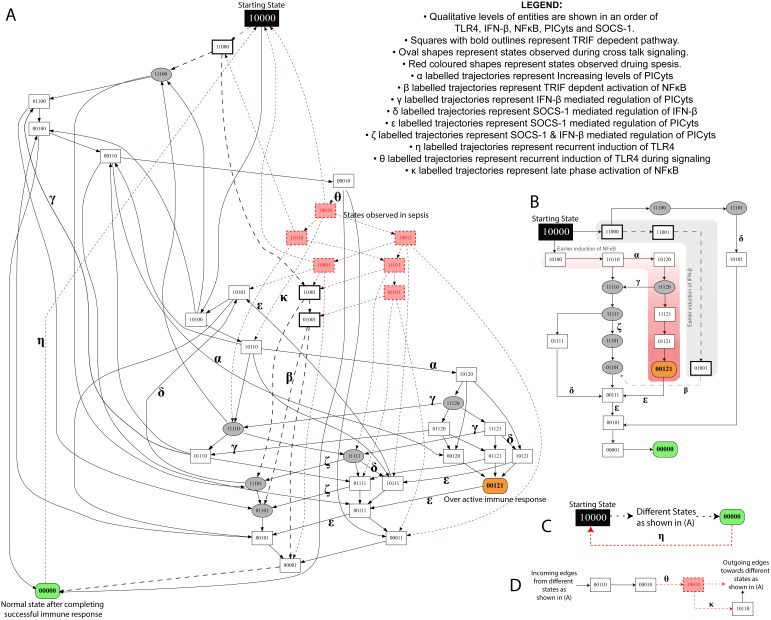
State graph of TLR4 and JAK/STAT signalling during non-septic and septic conditions. (A) Each node represents a particular state observed during signalling associated with non-septic and septic conditions. Integers “0”, “1” and “2” within the nodes represent qualitative levels of proteins in the order of TLR4, IFN-β, NFκB, PICyts and SOCS-1. Inactive entities are represented by integer “0” whereas active and overactive entities are represented by integers “1” and “2”, respectively. Nodes and trajectories, which were specifically observed during signalling dynamics associated with sepsis, are shown in red, whereas common nodes and trajectories found in both conditions are shown in black. Trajectories start from state “10000”, representing the activation of TLR4, and ultimately lead towards “00000”, which is a stable state in non-septic condition. On the other hand, a trajectory labelled with “η” from state “00000” to starting state “10000” results in a cyclic path during signalling dynamics associated with sepsis. MyD88 and TRIF dependent signalling are shown as black lines and dashed arrows, respectively. Nodes, which represent crosstalk of both signalling pathways i.e. IFN-β and NFκB with qualitative level “1” are presented in oval shapes. Arrows labelled with Greek small letters are used to represent trajectories associated with different signalling events (see legend in the figure). The conditions necessary to produce a state graph shown in the figure are given in Table 1. (B–D) specific states and trajectories which can possibly represent the complete state graph given in (A).

**Figure 4:**
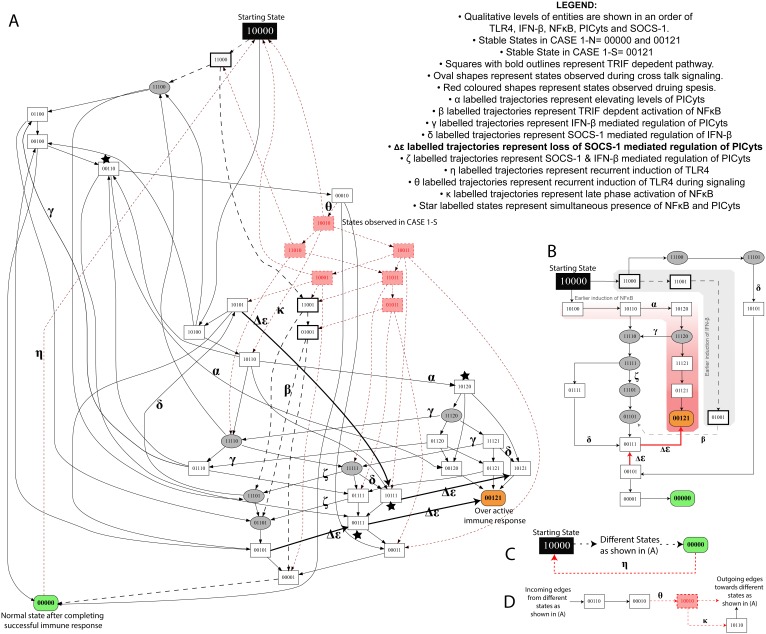
State graph of CASE 1-N and 1-S. (A) Each node represents a particular state observed during signalling associated with CASE 1-N and CASE 1-S. Integers “0”, “1” and “2” within the nodes represent qualitative levels of proteins in the order of TLR4, IFN-β, NFκB, PICyts and SOCS-1. Inactive entities are represented by integer “0” whereas active and overactive entities are represented by integers “1” and “2”, respectively. States and trajectories, which were specifically observed during signalling dynamics associated with CASE 1-S, are shown in red, whereas common states and trajectories found in both CASES are shown in black. Trajectories start from state “10000”, representing the activation of TLR4 and ultimately lead towards “00000” and “00121”, which are stable states in CASE 1-N. On the other hand, only one stable state “00121” was observed during signalling dynamics associated with CASE 1-S and a trajectory labelled with “η” from state “00000” to starting state “10000” results in cyclic path. Trajectories associated with loss of SOCS-1 mediated downregulation of PICyts in CASE 1-N and CASE 1-S are presented as bold arrows labelled with symbol “Δε”. Nodes are labelled with stars in which NFκB and PICyts were active simultaneously and have the probability to lead towards overactive immune response. MyD88 and TRIF dependent signalling are shown as black lines and dashed arrows, respectively. Nodes, which represent crosstalk of both signalling pathways i.e. IFN-β and NFκB with qualitative level “1” are presented in oval shapes. Arrows labelled with Greek small letters are used to represent trajectories associated with different signalling events (see legend in the figure). The conditions necessary to produce a state graph are shown in the figure are given in Table 3. (B–D) specific states and trajectories which can possibly represent the complete state graph given in (A).

**Figure 5:**
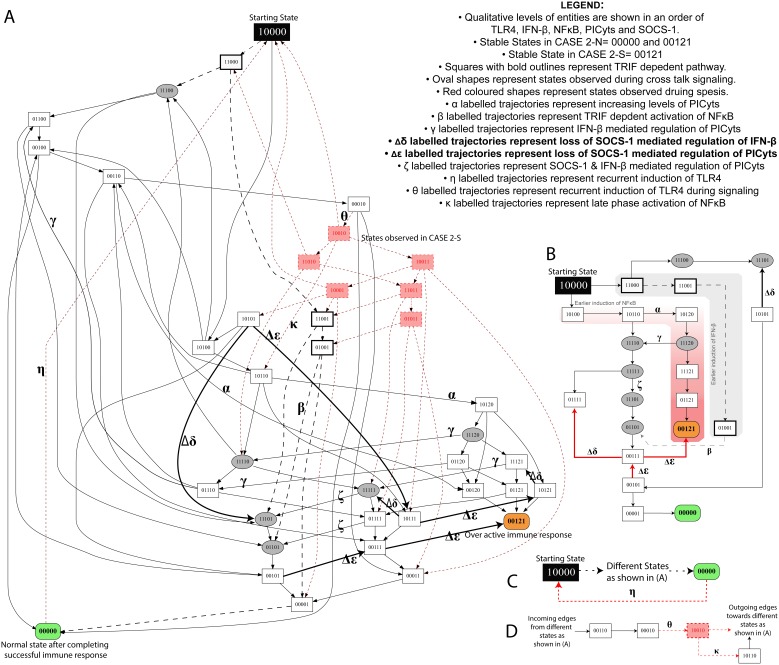
State graph of CASE 2-N and 2-S. (A) Each node represents a particular state observed during signalling associated with CASE 2-N and CASE 2-S. Integers “0”, “1” and “2” within the nodes represent qualitative levels of proteins in the order of TLR4, IFN-β, NFκB, PICyts and SOCS-1. Inactive entities are represented by integer “0” whereas active and overactive entities are represented by integers “1” and “2”, respectively. Nodes and trajectories, which were specifically observed during signalling dynamics associated with CASE 2-S, are shown in red, whereas common nodes and trajectories found in both CASES are shown in black. Trajectories start from state “10000”, representing the activation of TLR4 and ultimately lead towards “00000” and “00121”, which are stable states in CASE 2-N. On the other hand, only one stable state “00121” was observed during signalling dynamics associated with CASE 2-S and a trajectory labelled with “η” from state “00000” to starting state “10000” results in cyclic path. Trajectories associated with loss of SOCS-1 mediated downregulation of PICyts in CASE 2-N and CASE 2-S are presented as bold arrows labelled with symbol “Δε” whereas loss of SOCS-1 mediated downregulation of IFN-β are labelled with symbol “Δδ”. MyD88 and TRIF dependent signalling are shown as black lines and dashed arrows, respectively. Nodes, which represent crosstalk of both signalling pathways i.e. IFN-β and NFκB with qualitative level “1” are presented in oval shapes. Arrows labelled with Greek small letters are used to represent trajectories associated with different signalling events (see legend in the figure). The conditions necessary to produce a state graph shown in the figure are given in Table 3. (B–D) specific states and trajectories which can possibly represent the complete state graph given in (A).

**Figure 6:**
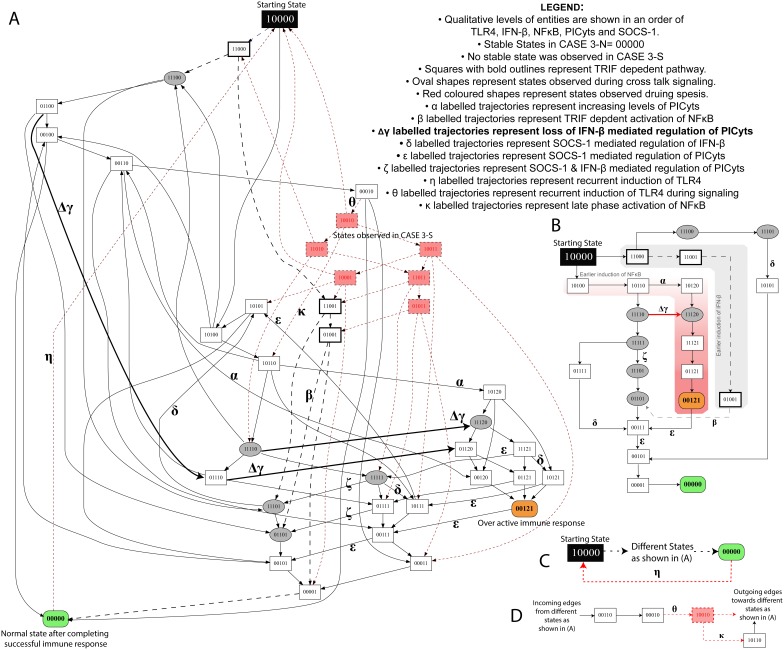
State graph of CASE 3-N and 3-S. (A) Each node represents a particular state observed during signalling associated with CASE 3-N and CASE 3-S. Integers “0”, “1” and “2” within the nodes represent qualitative levels of proteins in the order of TLR4, IFN-β, NFκB, PICyts and SOCS-1. Inactive entities are represented by integer “0” whereas active and overactive entities are represented by integers “1” and “2”, respectively. Nodes and trajectories, which were specifically observed during signalling dynamics associated with CASE 3-S, are shown in red, whereas common nodes and trajectories found in both CASES are shown in black. Trajectories start from state “10000”, representing the activation of TLR4, ultimately, lead towards “00000”, which is the stable state in CASE 3-N. On the other hand, a trajectory labelled with “η” from state “00000” to starting state “10000” results in a cyclic path during signalling dynamics associated with CASE 3-S. Trajectories associated with loss of IFN-β mediated downregulation of PICyts in CASE 3-N and CASE 3-S are presented as bold arrows labelled with symbol “Δγ”. MyD88 and TRIF dependent signalling are shown as black lines and dashed arrows, respectively. Nodes, which represent crosstalk of both signalling pathways i.e. IFN-β and NFκB with qualitative level “1” are presented in oval shapes. Arrows labelled with Greek small letters are used to represent trajectories associated with different signalling events (see legend in the figure). The conditions necessary to produce a state graph shown in the figure are given in Table 3. (B–D) specific states and trajectories which can possibly represent the complete state graph given in (A).

**Figure 7:**
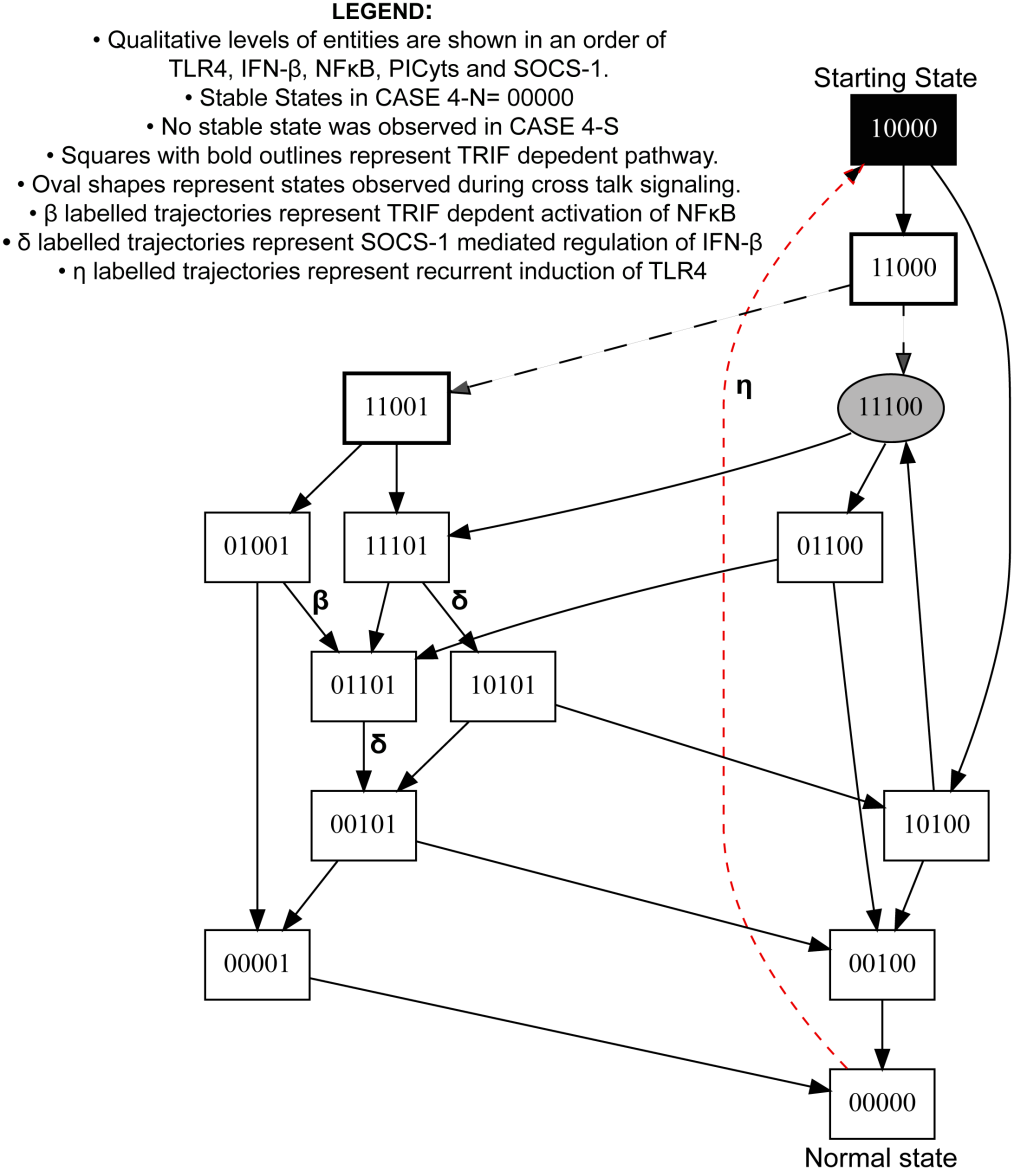
State graph of CASE 4-N and 4-S. Each node represents a particular state observed during signalling associated with CASE 4-N and CASE 4-S. Values “0” and “1” within the nodes represent qualitative levels of proteins in the order of TLR4, IFN-β, NFκB, PICyts and SOCS-1. Inactive entities are represented by integer “0” whereas active entities are represented by integer “1”. Trajectories, which were specifically observed during signalling dynamics associated with CASE 4-S, are shown in red, whereas common states and trajectories found in both CASES are shown in black. Trajectories start from state “10000”, representing the activation of TLR4, ultimately, lead towards “00000”, which is a stable state in CASE 4-N. On the other hand, a trajectory labelled with “η” from state “00000” to starting state “10000” results in a cyclic path during signalling dynamics associated with CASE 4-S. State “00121” which represents the immune response was absent in state graph and not shown in this figure. MyD88 and TRIF dependent signalling are shown as black lines and dashed arrows, respectively. Nodes, which represent crosstalk of both signalling pathways i.e. IFN-β and NFκB with qualitative level “1” are presented in oval shapes. Arrows labelled with Greek small letters are used to represent trajectories associated with different signalling events (see legend in the figure). The conditions necessary to produce a state graph shown in the figure are given in Table 3.

**Figure 8:**
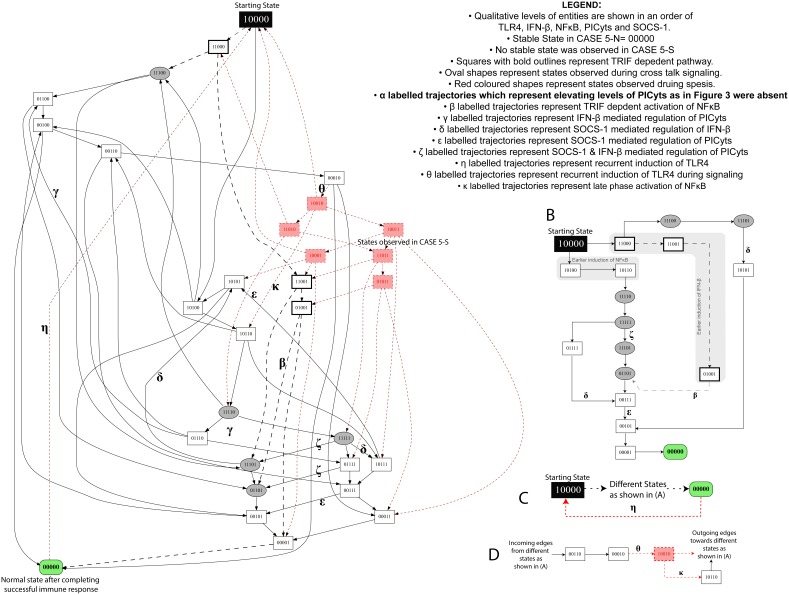
State graph of CASE 5-N and 5-S. (A) Each node represents a particular state observed during signalling associated with CASE 5-N and CASE 5-S. Integers “0” and “1” within the nodes represent qualitative levels of proteins in the order of TLR4, IFN-β, NFκB, PICyts and SOCS-1. Inactive entities are represented by integer “0” whereas active entities are represented by integer “1”. Nodes and trajectories, which were specifically observed during signalling dynamics associated with CASE 5-S, are shown in red, whereas common nodes and trajectories found in both CASES are shown in black. Trajectories start from state “10000”, representing the activation of TLR4, ultimately, lead towards “00000”, which is a stable state in CASE 5-N. On the other hand, a trajectory labelled with “η” from state “00000” to starting state “10000” results in a cyclic path during signalling dynamics associated with CASE 5-S. MyD88 and TRIF dependent signalling are shown as black lines and dashed arrows, respectively. Nodes, which represent crosstalk of both signalling pathways i.e. IFN-β and NFκB with qualitative level “1” are presented in oval shapes. Arrows labelled with Greek small letters are used to represent trajectories associated with different signalling events (see legend in the figure). The conditions necessary to produce a state graph shown in the figure are given in Table 3. (B–D) specific states and trajectories which can possibly represent the complete state graph given in (A).

One of the limitations of the kinetic logic approach is that it has been designed to analyse relatively small BRNs because of its scalability limitations [55]. For example, the TLR4 and JAK/STAT pathway as given in Figure 1 has 22 entities and on simulation its state graph would be composed of 6291456 states as compared to less than 50 states (Figures 3–8) generated by the reduced BRN (Figure 2).

The complexity of TLR4 and JAK/STAT pathways was reduced to make the BRN (shown in Figure 2) and resultant state graphs (Shown in Figures 3–8) tractable. Starting with the complete signalling pathways of TLR4 and JAK/STAT, adopted from previous experimental studies and databases associated with biological signalling [28]–[33], the BRN was reduced iteratively by following the strategies of Naldi *et. al.* [69] and Assieh *et. al.* [70]. Briefly, one such example is if an entity X1 activates another entity X2, which in turn activates X3 such that X3 inhibits X1, then we can omit X2 and represent this relation as a simple feedback loop where X1 activates X3 and X3 inhibits X1. In the process of reduction, the behaviour of the removed entity X2 was preserved implicitly in the activation of X3 by X1 to account for the related interactions and their effects on the target nodes. Another example is given in the Figure 3.3 of the study by Assieh *et al.* [70], where a network of 13 proteins (Figure 3.3a) is reduced to 3 proteins (Figure 3.3b) using the reduction rules. Similarly in the study of Naldi *et.al.* [69], Figure 2 is presenting another example of the BRN reduction.

### Discrete Modelling of the BRN

GENOTECH (provided at http://code.google.com/p/genotech/downloads/list. Steps necessary to model a BRN in GENOTECH have been described in [57]) and GINSIM (documentation and software for modelling BRNs in GINSIM are available at www.ginsim.org) [71] facilitated the construction of qualitative model of BRN (Figure 2) according to Thomas’ formalism. Modelling of the BRN was performed by inserting the required entities as nodes and drawing the corresponding interactions as edges. Threshold levels and logical parameters for each entity were adjusted as discussed below. Using asynchronous strategy, the results were produced in the form of state graphs composed of states and trajectories consisting of cycling paths and paths diverging towards stable states. These state graphs were used to study the dynamics of the BRN. GENOTECH files and equivalents in GINSIM format have been provided as Files S1–S24, for each condition discussed in results.

### Threshold values of each entity in the BRN

According to the Definition 2, the threshold level 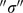 is a positive integer, which represents the minimum qualitative level of an entity necessary to activate or inhibit its target entities. In contrast to the Boolean logic, kinetic logic (multivalued) permits the use of threshold level 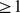 [61], [72]. The threshold values, which can be used, depend upon the outgoing edges from any entity. These values range from “1” to the number of outgoing edges from an entity. The reason for multivalued formalism is that a particular entity can activate or inhibit its target entities at different activation levels and thus require more than one threshold level to perform its role as an activator or inhibitor. For example SOCS-1 has been implicated for its inhibitory role in TLR4, NFκB, IFN-β and PICyts mediated signalling [48] but the specific expression levels of SOCS-1 which are necessary to inhibit all of these four entities in the presence of other resources (activators or inhibitors) are not reported. Therefore, according to Thomas’ formalism, the threshold levels of SOCS-1 for its interaction with these four entities can be within the range 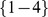. In order to keep the model simple, the threshold levels of SOCS-1 were set at level “1”. Similarly, for other entities, including TLR4, IFN-β and NFκB, the threshold levels were set at “1”. Only PICyts mediated activation of NFκB-JAK/STAT was set at “2”. Therefore, PICyts was supposed to activate SOCS-1 at the activation level “1” and NFκB-JAK/STAT at activation level “2”. Threshold level “2” was used in speculation that PICyts activate the products of NFκB-JAK/STAT signalling pathway after reaching a certain qualitative threshold level, which may differ from its other actions such as activation of SOCS-1 [73]–[75].

### Types of interaction

The entities in a BRN may represent proteins or genes, which can interact with each other. Depending upon the threshold levels, entities can either activate or inhibit other entities termed as evolving or target entities (see Definition 2 where 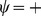 for activators and 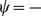 for inhibitors). As shown in the BRN (Figure 2), TLR4, IFN-β, NFκB and PICyts are the entities, which can either activate each other or SOCS-1 depending upon their threshold levels. According to formalism, whenever the activation of these entities will reach to their threshold levels, generally taken as “1”, then successors of these entities will also be activated. This relationship can be depicted by sigmoidal graph presented as Figure 5A in our previous study [57]. It can be seen that an activator below a threshold level (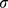) slightly affects the activation level of its target entity. However, as soon as the activator achieves its threshold level, then the target entity also reaches to active state where it can perform its further interactions. In other words, whenever entity has threshold level <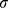 then it cannot activate its target entities but when threshold level of an entity ≥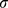 then it can activate its target entities. In this scenario, the entities are termed as positive regulators or activators when they activate other entities during the dynamics of the BRN shown as state graphs.

On the other hand, IFN-β, NFκB and SOCS-1 can inhibit either each other or TLR4 and PICyts. These entities are termed as negative regulators or inhibitors and process is termed as downregulation or inactivation. Entities which can inhibit evolving entities also depend upon their threshold levels. The effect of inactivation is also of sigmoidal nature and is shown as Figure 5B in our previous study [57].

### Logical parameters of each entity in the BRN

Logical parameters have been described by using the relation 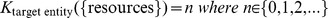. Where resources are those entities of BRN, which are connected with evolving or target entity. These resources can be either activators or inhibitors depending upon their presence or absence, respectively, during a particular state. Activators were taken as resources when they were present in a particular state. On the other hand, inhibitors were taken as resources only when they were absent during a particular state.

According to the formalism, the possible number of logical parameters, which we have to define for each evolving entity depends upon the number of resources. If the number of resource is one, then the possible number of logical parameters, which we have to define, will be two. This relation can be shown as a power set of the set of regulators (set of activators and inhibitors) of an entity. Therefore, each logical parameter corresponds to one element of the power set. In accordance with the René Thomas’ formalism, the total number of logical parameters for TLR4, IFN-β, NFκB, PICyts and SOCS-1 are 4, 8, 16, 8 and 4, respectively (Table 1).

**Table 1:** Logical parameters used for each entity in modelling of non-septic TLR4 and JAK/STAT signalling using the BRN shown in Figure 2.

S.No.	Logical Parameters
1	K_TLR4_({}) = 0
2	K_TLR4_({*NFκB*}) = 0
3	K_TLR4_({*SOCS-1*}) = 0
4	K_TLR4_({*SOCS-1, NFκB*}) = 0
5	K_NFκB-JAK/STAT_({}) = 0
6	K_NFκB-JAK/STAT_({*TLR4*}) = 1
7	K_NFκB-JAK/STAT_({*IFN-β*}) = 1
8	K_NFκB-JAK/STAT_({*PICyts*}) = 1
9	K_NFκB-JAK/STAT_({*TLR4*, *IFN-β*}) = 1
10	K_NFκB-JAK/STAT_({*TLR4, PICyts*}) = 1
11	K_NFκB-JAK/STAT_({*IFN-β, PICyts*}) = 1
12	K_NFκB-JAK/STAT_({*TLR4, PICyts, IFN-β*}) = 1
13	K_NFκB-JAK/STAT_({*TLR4, SOCS-1*}) = 1
14	K_NFκB-JAK/STAT_({*IFN-β, SOCS-1*}) = 1
15	K_NFκB-JAK/STAT_({*PICyts, SOCS-1*}) = 1
16	K_NFκB-JAK/STAT_({*TLR4, IFN-β, SOCS-1*}) = 1
17	K_NFκB-JAK/STAT_({*TLR4, PICyts, SOCS-1*}) = 1
18	K_NFκB-JAK/STAT_({*PICyts, IFN-β, SOCS-1*}) = 1
19	K_NFκB-JAK/STAT_({*SOCS-1*}) = 0
20	K_NFκB-JAK/STAT_({*SOCS-1, TLR4, IFN-β, PICyts*}) = 1
21	K_PICyts_({}) = 0
22	K_PICyts_({*NFκB*}) = 0
23	K_PICyts_({*IFN-β*}) = 0
24	K_PICyts_({*SOCS-1*}) = 0
25	K_PICyts_({*NFκB, IFN-β*}) = 0
26	K_PICyts_({*NFκB, SOCS-1*}) = 0
27	K_PICyts_({*IFN-β, SOCS-1*}) = 0
28	K_PICyts_({*NFκB, IFN-β, SOCS-1*}) = 2
29	K_SOCS-1_({}) = 0
30	K_SOCS-1_({*PICyts*}) = 1
31	K_SOCS-1_({*IFN-β*}) = 1
32	K_SOCS-1_({*PICyts, IFN-β*}) = 1
33	K_IFN-β_({}) = 0
34	K_IFN-β_({*TLR4*}) = 0
35	K_IFN-β_({*SOCS-1*}) = 0
36	K_IFN-β_({*NFκB*}) = 0
37	K_IFN-β_({*TLR4, SOCS-1*}) = 1
38	K_IFN-β_({*TLR4, NFκB*}) = 1
39	K_IFN-β_({*SOCS-1, NFκB*}) = 0
40	K_IFN-β_({*TLR4, SOCS-1, NFκB*}) = 1

Each logical parameter has been discussed in detail in File S26.

The value 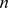 for each logical parameter was unknown a priori, and was computed using the Selection of Models of Biological Networks (SMBioNet) tool [76]–[78]. Briefly, this tool is based on the kinetic logic formalism of René Thomas that takes a BRN with unspecified parameters and Computational Tree Logic (CTL) [79] formulae of the form 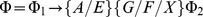 representing a specific biological behaviour (observation). In the formula 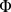, the path quantifier 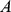 or 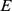 governs if a specific property should hold in all trajectories (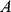) originating from a current state or in at least one trajectory (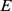). Whereas, the state quantifiers 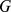, 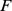 and 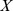 govern if a property should hold in all states (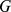), in a future state (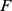), or in the immediate successor state (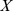) in a trajectory (path); and finally 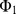 and 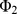 represent the Boolean logic formulae representing the initial expression levels and the observed expression levels of the entities, respectively. In the formula 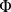, the symbol “
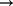
” represents the Boolean implication operator. For example, the formula 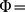
((TLR4 = 1 AND IFN-β = 0 AND NFκB = 0 AND PICyts = 0 AND SOCS-1 = 0) 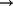 *EF*(NFκB = 1)) is a CTL formula where 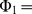 (TLR4 = 1 AND IFN-β = 0 AND NFκB = 0 AND PICyts = 0 AND SOCS-1 = 0) representing the initial expression levels of entities (TLR4 is currently active and others are inactive), and *EF*(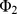) with 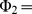
(NFκB = 1) represents the observation that NFκB eventually activates. In this example, AND is the Boolean conjunction operator. The CTL encoded observed biological behaviours from the literature pertaining to the TLR4 and JAK/STAT pathway are given in Table 2. The input and output of the SMBioNet are provided in File S25.

**Table 2:** Biological observations and concerned references from previous literature which were used to generate the CTL formula as given as input to SMBioNet.

S#	Biological observations	CTL formula in SMBioNet
1	Once TLR4 gets activated, it will thenactivate the downstream signalingin response to infection, which eventuallyleads to the induction of NFκB and IFN-β[103],[112]–[116].	((TLR4 = 1&IFNb = 0&NFkB = 1&PICyts = 0&SOCS1 = 0)->EF(TLR4 = 1&IFNb = 1&NFkB = 1))
2	After a successful immuneresponse or clearance of infection,all the entities will bedownregulated [20], [25], [117].	((TLR4 = 1&IFNb = 0&NFkB = 0&PICyts = 0&SOCS1 = 0)->EF(AG(TLR4 = 0&IFNb = 0&NFkB = 0&PICyts = 0&SOCS1 = 0)))

Value “0” represents that evolving entity deactivates in the presence of its resources, whereas a value “1” represents activation of an entity. However, the logical parameter 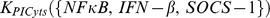 was with a value “2” depending upon the threshold level of PICyts for the induction of JAK-STAT pathway as discussed earlier. The values of these computed logical parameters were validated by previous literature. An informal description of the logical parameters with relevant evidences has been provided as File S26 that form the basis for using a specific value for each logical parameter given in Table 1.

These logical parameters were further validated by the proved conjectures [a positive feedback circuit (respectively negative feedback circuit) is a necessary condition for multistationarity (respectively homeostasis). In a BRN a positive feedback circuit (respectively negative feedback circuit) is the one which contains even (respectively odd) number of negative interactions] of René Thomas [55] and biologically observed stable states. Logical parameters given in Table 1 were finally used to study the dynamics of the BRN (Figure 2) in the form of state graphs. These parameters have also been shown as tendency graphs in Figures S9–S13 using sigmoidal graphs among evolving entities and their resources.

### Modelling of interventions

Models with interventions were derived by removing one or more of the interactions of IFN-β, SOCS-1, NFκB and PICyts present in the BRN (Figure 2). Associated logical parameters were also changed or removed to maintain the integrity of each model (Table 3). These interventions (discussed as CASES) were used to observe their impact on the signalling events (effects on the cyclic paths and stable states during signalling modelled for septic and non-septic) and for comparison with non-intervened or intact models. All the models which are discussed in this study have been provided as files in GENTOCH (Files S1–S12) and GINSIM (Files S13–S24) formats.

**Table 3:** Intervened signalling.

CASE	EvolvingEntity	Targetentity/ies	Removedparameters	Changedparameters	Removededge/s in Figure 2.	ProducedStable states
1-N	SOCS-1	PICyts	K_PICyts_({*SOCS-1*}) = 0,K_PICyts_({*NFκB, SOCS-1*}) = 0,K_PICyts_({*NFκB, IFN-β, SOCS-1*}) = 0	K_PICyts_({*NFκB, IFN-β*}) = 2	SOCS-1 mediateddownregulation of PICyts	00000 & 00121
1-S	SOCS-1	PICyts	K_PICyts_({*SOCS-1*}) = 0,K_PICyts_({*NFκB, SOCS-1*}) = 0,K_PICyts_({*NFκB, IFN-β, SOCS-1*}) = 0	K_PICyts_({*NFκB, IFN-β*}) = 2,K_TLR4_({*SOCS-1, NFκB*}) = 1	SOCS-1 mediateddownregulation of PICytsduring recurrent TLR4 signalling	00121
2-N	SOCS-1	PICyts &IFN-β	K_PICyts_({*SOCS-1*}) = 0,K_PICyts_({*NFκB, SOCS-1*}) = 0,K_PICyts_({*NFκB, IFN-β,* *SOCS-1*}) = 0,K_IFN-β_({*SOCS-1*}) = 0,K_IFN-β_({*TLR4, SOCS-1*}) = 1,K_IFN-β_({*SOCS-1, NFκB*}) = 0,K_IFN-β_({*TLR4, SOCS-1, NFκB*}) = 1	K_PICyts_({*NFκB, IFN-β*}) = 2	SOCS-1 mediateddownregulation ofIFN-β and PICyts	00000 & 00121
2-S	SOCS-1	PICyts &IFN-β	K_PICyts_({*SOCS-1*}) = 0,K_PICyts_({*NFκB, SOCS-1*}) = 0,K_PICyts_({*NFκB, IFN-β, SOCS-1*}) = 0,K_IFN-β_({*SOCS-1*}) = 0,K_IFN-β_({*TLR4, SOCS-1*}) = 1,K_IFN-β_({*SOCS-1, NFκB*}) = 0,K_IFN-β_({*TLR4, SOCS-1, NFκB*}) = 1	K_PICyts_({*NFκB, IFN-β*}) = 2,K_TLR4_({*SOCS-1, NFκB*}) = 1	SOCS-1 mediateddownregulation of IFN-βand PICyts during recurrentTLR4 signalling	00121
3-N	IFN-β	PICyts	K_PICyts_({*IFN-β*}) = 0,K_PICyts_({*IFN-β, NFκB*}) = 0,K_PICyts_({*IFN-β, SOCS-1*}) = 0,K_PICyts_({*NFκB, IFN-β, SOCS-1*}) = 2	K_PICyts_({*NFκB, SOCS-1*}) = 2	IFN-β mediateddownregulation of PICyts	00000
3-S	IFN-β	PICyts &SOCS-1	K_PICyts_({*IFN-β*}) = 0,K_PICyts_({*IFN-β, NFκB*}) = 0,K_PICyts_({*IFN-β, SOCS-1*}) = 0,K_PICyts_({*NFκB, IFN-β, SOCS-1*}) = 2	K_PICyts_({*NFκB, SOCS-1*}) = 2,K_TLR4_({*SOCS-1, NFκB*}) = 1	IFN-β mediateddownregulation PICyts duringrecurrent TLR4 signalling	00000
4-N	NFκB	PICyts	K_PICyts_({*NFκB*}) = 0,K_PICyts_({*NFκB, IFN-β*}) = 0,K_PICyts_({*NFκB, SOCS-1*}) = 0,K_PICyts_({*NFκB, IFN-β, SOCS-1*}) = 2	-	NFκB mediatedinduction of PICyts	00000
4-S	NFκB	PICyts	K_PICyts_({*NFκB*}) = 0,K_PICyts_({*NFκB, IFN-β*}) = 0,K_PICyts_({*NFκB, SOCS-1*}) = 0,K_PICyts_({*NFκB, IFN-β, SOCS-1*}) = 2	K_TLR4_({*SOCS-1, NFκB*}) = 1	NFκB mediated inductionof PICyts during recurrentTLR4 signalling	00000
5-N	PICyts	NFκB-JAK/STAT	K_NFκB-JAK/STAT_({*PICyts*}) = 1,K_NFκB-JAK/STAT_({*TLR4, PICyts*}) = 1,K_NFκB-JAK/STAT_({*IFN-β, PICyts*}) = 1,K_NFκB-JAK/STAT_({*TLR4, PICyts, IFN-β*}) = 1,K_NFκB-JAK/STAT_({*PICyts, SOCS-1*}) = 1,K_NFκB-JAK/STAT_({*TLR4, PICyts, SOCS-1*}) = 1,K_NFκB-JAK/STAT_({*PICyts, IFN-β, SOCS-1*}) = 1,K_NFκB-JAK/STAT_({*SOCS-1, TLR4, IFN-β,* *PICyts*}) = 1	-	PICyts mediated inductionof JAK/STAT signalling	00000
5-S	PICyts	NFκB-JAK/STAT	K_NFκB-JAK/STAT_({*PICyts*}) = 1,K_NFκB-JAK/STAT_({*TLR4, PICyts*}) = 1,K_NFκB-JAK/STAT_({*IFN-β, PICyts*}) = 1,K_NFκB-JAK/STAT_({*TLR4, PICyts, IFN-β*}) = 1,K_NFκB-JAK/STAT_({*PICyts, SOCS-1*}) = 1,K_NFκB-JAK/STAT_({*TLR4, PICyts, SOCS-1*}) = 1,K_NFκB-JAK/STAT_({*PICyts, IFN-β, SOCS-1*}) = 1,K_NFκB-JAK/STAT_({*SOCS-1,* *TLR4, IFN-β, PICyts*}) = 1	K_TLR4_({*SOCS-1, NFκB*}) = 1	Loss of PICyts mediatedinduction of JAK/STATsignalling duringrecurrent TLR4 induction.	00000

Different CASES have been presented with respective changes in parameters. Changes presented here in each CASE accompanied other logical parameters described in Table 1 to model each CASE.

## Results

Different perspectives of the TLR4 and JAK/STAT signalling were studied by simulating septic and non-septic conditions, both in the presence and absence of specific interactions among entities. The devised logical parameters for all entities (Table 1) were used to model non-septic dynamics of TLR4 and JAK/STAT signalling shown as BRN in Figure 2. Changes in specific logical parameters by removing respective edges or interactions between the entities as given in Figure 2 were used to model septic and intervened signalling (Table 3). State graphs shown in Figures 3–8 represent signalling events or dynamics of different perspectives of BRN discussed in detail below. Qualitative levels (0, 1 or 2) of TLR4, IFN-β, NFκB, PICyts and SOCS-1 represent qualitative states, which are shown as nodes, whereas trajectories represent possible progress or evolution paths of entities depending upon the logical parameters and qualitative threshold levels (Figures 3–8). In each state graph, state “
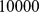
” represents the starting state (activation of TLR4 as first signal) whereas states of “
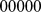
” and “
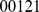
” represents the downregulated and overactive immune responses, respectively.

### Signalling in non-septic case

All the logical parameters used in modelling the non-septic signalling are defined in Table 1 and shown as dummy tendency graphs in Figures S9–S13. Model related to non-septic condition has been provided as File S1. Logical parameters devised for each entity were based on the experimental findings, but the incorporation of several experimental findings as a single rule for evolving entity were devised as discussed in the methods. Logical parameters were devised in such a way that after the production of overactive immune response (state “
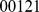
”), the dynamics of the BRN should reach to a stable state “
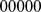
” representing the downregulation of immune response. Simulation of non-septic model led to the generation of a state graph shown in Figure 3.

Complete TLR4 mediated induction of TRIF and MyD88 adaptor proteins are represented by the induction of IFN-β (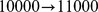) and NFκB (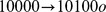), respectively. According to the previous experimental studies, MyD88 dependent pathway is induced in preference to the TRIF dependent pathway [80], [81]. In the state graph, it can be noticed that the induction of PICyt was achieved only in those trajectories in which NFκB mediated signalling was activated in preference to IFN-β. On the other hand, chances for the induction of PICyt were found comparatively lower in the presence of earlier induced IFN-β. Probably, this may create a platform for immediately required immune response against the pathogen. States, which represent a crosstalk mechanism of MyD88 and TRIF dependent signalling, are shown within the oval shapes. Downstream to these states, the presence of SOCS-1 and/or IFN-β compelled the system towards immunocompromised states (states with level “0” or “1” of PICyts). Most of the crosstalk states were observed during the activated TLR4. However, in the absence of TLR4, TRIF induced activation of NFκB (shown as 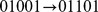) can be specifically observed in trajectories labelled with “β” in Figure 3. Correlated with previous experimental study, this transition was produced nearly at the end of dynamics and triggered the late phase induction of NFκB mediated proinflammatory immune responses [36].

During dynamics of the BRN, trajectories (labelled with “α” in Figure 3) were found most important for the over activation of PICyts which include (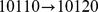) and (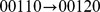). Both of these trajectories were associated with the absence of SOCS-1 and IFN-β along with elevated levels of PICyts (shown by level “2”). Moreover, both of these trajectories may represent the importance of TLR4 and JAK/STAT mediated induction of inflammatory responses.

The presence of IFN-β and/or SOCS-1 at different levels in the state graph were found necessary to downregulate the levels of PICyts. After the expression of PICyts, recursive action of IFN-β and then SOCS-1 maintained the homeostasis of the immune system by downregulating the levels of PICyts. After achieving the hyperinflammatory state “
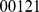
”, dynamics of the BRN were led towards stable state “
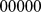
”, which represents the downregulation of the immune system. Trajectories labelled with “γ” in Figure 3 highlights the role of IFN-β mediated downregulation of PICyts. The presence of IFN-β reduced the chances for induction of PICyts (trajectories downstream of state “
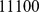
”) and delayed effective immune response. However, in the absence of IFN-β, fate of system was shifted towards NFκB mediated induction of PICyts.

Trajectories labelled with “δ” and “ε” in Figure 3 represent SOCS-1 mediated downregulation of IFN-β and PICyts, respectively. Generally, the behaviour of SOCS-1 was found to downregulate the levels of IFN-β in preference to PICyts. Indirectly, SOCS-1 allowed PICyts to higher expression levels in the system and then regulated the same. Some of the trajectories (labelled with “ζ” in Figure 3) in the state graph infer the combination of SOCS-1 and IFN-β mediated downregulation of PICyts and observed mostly during crosstalk of MyD88 and TRIF dependent pathways, which led the dynamics towards downregulated immune response. In this setting, it can be assumed that SOCS-1 may allow extra time for the induction of PICyts so that the immune system can cope up with the pathogens. However, subsequent SOCS-1 mediated inhibition of PICyts may implicate the decrease in damage to the host by its exacerbating immune response.

The presence of cyclic paths, termed as strongly connected components (SCC)-1 and SCC-2, in the state graph can be seen in Figure S1 and S2, respectively. SCC-1 highlights the importance of states “
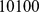
” and “
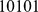
” (Figure S1). The cyclic path between these two states represent the recurrent activation of PICyts in non-septic TLR4 signalling with subsequent downregulation. Due to the presence of IFN-β and SOCS-1, system cycled through trajectory “
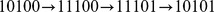
” and produced sustained immunosuppression. On the other hand, SCC-2 is a representation of the cycle among states produced after the induction of JAK/STAT pathway (Figure S2). During this cycle, only SOCS-1 mediated downregulation of PICyts can be seen in absence of IFN-β.

### Signalling in sepsis

The continuous presence of pathogens or recurrent infections can ignite rigorous immune responses in the host’s body [82]. Previous experimental studies suggested the role of recurrent induction of TLRs in persistent infections and sepsis [83], [84]. In this study, continuous induction of TLR4 was the only difference between the set of logical parameters used for model septic and non-septic signalling. Therefore, recurrent induction of TLR4, represented by logical parameter (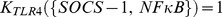), was used with other logical parameters of entities given in Table 1 to model the sepsis related signalling. Model related to sepsis has been provided as File S2. Dynamics of the BRN were studied in the form of a state graph, which was merged in Figure 3 for the purpose of comparison. In Figure 3, red highlighted states in squares and dotted trajectories represent the additional states and trajectories produced during septic signalling whereas common states and trajectories both in non-septic and septic systems are shown in black.

Unlike non-septic TLR4 and JAK/STAT signalling, new events of recurrent TLR4 induction (trajectories 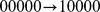, labelled with “η” and 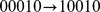, labelled with “θ”) and the absence of stable state “
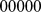
” were observed as characteristics of sepsis. Overall, two phases of signalling were observed in the state graph shown in Figure 3. The first phase of signalling was comparable to non-septic signalling whereas the second phase of signalling represented a late phase of signalling dynamics produced due to repetitive TLR4 induction. In this phase, TLR4 was re-induced during pre-existing levels of PICyts (trajectory 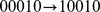 labelled with “θ”). Later to which, influence of IFN-β or SOCS-1 tolerated the levels of PICyts with subsequent degradation of PICyts. Comparatively, most of the states in the late phase of dynamics represented immunosuppression due to the presence of both IFN-β and SOCS-1.

Induction of NFκB as in the trajectory (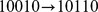 labelled with “κ” in Figure 3) was the only trajectory, which strengthened the levels of PICyts and led the system to overactive immune response (state “00121”) in the late phase of signalling. However, all the trajectories ultimately led the system towards downregulated immune response (state “
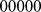
”). Subsequent activation of the new round of TLR4 mediated signalling after the state “
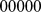
” represented the recurrent induction of TLR4 (transition labelled with “η”) in absence of any other downstream proteins. In summary, the phenomenon of oscillation was present representing activation and inactivation of PICyts during complete dynamics related to the condition of sepsis along with the suppressed expression levels of PICyts in late phase of signalling dynamics of the BRN.

### Interventions in signalling

Mutations and/or therapeutic interventions can change the role of resources with subsequent changes in the dynamics of the BRN (see Definition 4 in methods section). Interventions were modelled by removing one or more interactions associated with any entity present in the BRN (Figure 2) to reproduce mutations or therapeutic interventions. The effects of interventions in IFN-β, SOCS-1, NFκB and PICyts mediated signalling were compared both in septic and non-septic signalling to elaborate their importance in the dynamics of the BRN. These interventions are discussed as “CASES” and changes in logical parameters are mentioned in Table 3. Other possible interventions, given in Table S1, were also analysed to observe their overall effects on the system in terms of stable states produced by each type of intervention.

#### CASE 1 (Intervention in SOCS-1 mediated downregulation of PICyts)

Intervention in SOCS-1 mediated downregulation of PICyts during non-septic signalling is discussed as CASE 1-N whereas in case of sepsis, it is discussed as CASE 1-S. Modelling of CASE 1-N was performed using the logical parameters given in Table 1 except with some changes as given in Table 3. The model is provided as File S3. Figure 4 represents the state graph produced due to the simulation of CASE 1-N. In this case, two stable states were observed including downregulated or normal (state “
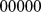
”) and overactive PICyt levels (state “
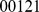
”). The overall dynamics of the system were found comparable to the non-septic signalling (as shown in Figure 3) but some trajectories involving SOCS-1 mediated inhibition of PICyts were different (trajectories labelled with “Δε” in Figure 4). In those trajectories, SOCS-1 mediated inhibition of PICyts was suppressed and permitted continuous activation of PICyts with subsequent induction of JAK/STAT pathway (stable state “
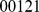
”). These trajectories were also found opposite in directions from those observed in non-septic signalling (trajectories labelled with “ε” in Figure 3); where SOCS-1 mediated downregulation of PICyts led the system towards stable state “
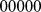
”. IFN-β mediated downregulation of PICyts in CASE 1-N was found ineffective to reduce the levels of PICyts because IFN-β performed its inhibitory role in the earlier part of the dynamics (trajectories labelled with “γ” in Figure 4). Moreover, PICyts was capable enough to induce SOCS-1 mediated inhibition of IFN-β which results in its continuous inactivated state during later stages of the signalling. Thus higher levels of PICyts led the system to overactive immune response (trajectories labelled with “δ” in Figure 4).

It was observed that the system evolved mostly towards stable state “
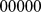
” in absence of activated TLR4. This condition was true except for those states in which NFκB and PICyts were present simultaneously in the system (downstream signalling dynamics after states 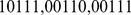 and 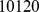 labelled with stars in Figure 4). All of these states had a higher probability to produce overactive immune state (stable state “00121”) in the system. The presence of homeostasis in the state graph was found comparable to SCC-1 (Figure S1) whereas SOCS-1 mediated downregulation and homeostasis in PICyts levels was absent.

States and trajectories related to CASE 1-S were incorporated in Figure 4. Model of CASE 1-S is provided as File S4. Overall, dynamics of the BRN in CASE 1-S were found similar to the CASE 1-N but some of the trajectories involving re-activation of TLR4 were found different (trajectory labelled with “η” in Figure 4). Instead of two stable states as seen in CASE 1-N, only one stable state “00121” was found in CASE 1-S. New events of recurrent TLR4 induction (trajectories 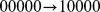, labelled with “η” and 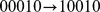, labelled with “θ”) were comparable to signalling in sepsis without any intervention as shown in Figure 3. Unlike signalling in sepsis without any interventions, SOCS-1 did not play its part in late phase signalling dynamics. Induction of IFN-β in the late phase of septic signalling was also found ineffective to attenuate the overactive immune responses in the absence of SOCS-1.

#### CASE 2 (Intervention in SOCS-1 mediated downregulation of IFN-β and PICyts)

One of the important effects on the dynamics of the BRN would be the complete loss of SOCS-1 mediated inhibition of IFN-β and PICyts so that their levels could be elevated. To evaluate this, intervention in SOCS-1 mediated inhibition of IFN-β and PICyts was executed. Intervention in non-septic state is discussed as CASE 2-N, whereas this intervention in case of sepsis is discussed as CASE 2-S. Dynamics of the BRN in these CASES are shown as a state graph in Figure 5. Modelling was performed using the logical parameters given in Table 1 along with certain modifications (Table 3). Moreover, respective edges, which represent SOCS-1 mediated inhibition of IFN-β and PICyts in Figure 2, were also removed.

Model of CASE 2-N is provided as File S5. The dynamics of the BRN produced in CASE 2-N were found comparable to non-septic case (Figure 3) except those trajectories which reflected SOCS-1 mediated inhibition of IFN-β and PICyts (trajectories labelled with “Δδ” and “Δε”, respectively in Figure 5). Trajectories labelled with “Δδ” and “Δε” were found opposite in the direction as compared to their counterparts in non-septic signalling (Figure 3). Stable states “
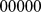
” and “
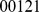
” were comparable to those found in CASE 1-N. Similar to other state graphs discussed above, the simultaneous presence of NFκB-JAK/STAT and PICyts led the trajectories mostly towards elevated levels of PICyts even in the absence of SOCS-1 mediated inhibition of IFN-β. Homeostatic downregulation was seen comparable to SCC-1 (Figure S1), whereas another homeostasis produced during non-intervened signalling (SCC-2) was abolished.

Signalling in CASE 2-S was incorporated into the Figure 5 and its model is provided as File S6. In this CASE, most of the states and trajectories associated with sepsis were found comparable to those in CASE 2-N. Only one stable state “
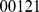
” was found in CASE 2-S, whereas another stable state “00000” led the dynamics of the BRN towards recurrent induction of TLR4 like other sepsis related dynamics in this study. New events of recurrent TLR4 induction (trajectories 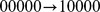, labelled with “η” and 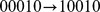, labelled with “θ”) were similar to the sepsis related non-intervened dynamics of the BRN (Figure 3). However, in this CASE, SOCS-1 could not play its inhibitory role in late phase signalling dynamics. Moreover, interventions in SOCS-1 mediated inhibition of IFN-β produced similar results as seen in CASE 1-S due to the activated levels of TLR4.

#### CASE 3 (Intervention in IFN-β mediated downregulation of PICyts)

Intervention in IFN-β mediated inhibition of PICyts during non-septic and septic conditions are discussed as CASE 3-N and CASE 3-S, respectively. These CASES were used to evaluate the dynamics of the BRN and consequential immune responses in the absence of IFN-β mediated inhibition of PICyts. Intervention was derived by removing the inhibitory edge from IFN-β to PICyts as given in Figure 2. Modelling was performed using the logical parameters given in Table 1 with some exceptions given in Table 3 for CASE 3-N and CASE 3-S. Models of CASE 3-N and CASE 3-S are also provided as File S7 and File S8, respectively). A state graph of CASE 3-N is shown in Figure 6.

Only one stable state “
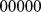
” was observed during the dynamics of the BRN associated with CASE 3-N. It was observed that IFN-β mediated inhibition of PICyts during earlier phase of signalling was abolished from the system (trajectories labelled with “Δγ” in Figure 6). This interaction was speculated to resist the elevated levels of PICyts as seen above in Figures 3–5. However, the elevated levels of PICyts were downregulated by SOCS-1 when IFN-β was unable to inhibit PICyts.

Homeostatic signalling in CASE 3-N (SCC-3) was similar to that produced during the dynamics of non-septic model (SCC-1), however, trajectories were slightly shifted towards elevated levels of PICyts (Figure S3). Homeostasis during overactive PICyts (state “00121”) was observed in the presence of SOCS-1 (SCC-4 and SCC-5 shown in Figure S4 and Figure S5, respectively), which represents that even in the absence of IFN-β, SOCS-1 cater the inhibition of PICyts.

Signalling in CASE 3-S was integrated in the state graph produced by CASE 3-N (Figure 6). Most of the trajectories and nodes were found common except those which involved re-activation of TLR4, as discussed above. Late phase IFN-β mediated downregulation of PICyts during septic signalling was not found.

#### CASE 4 (intervention in NFκB mediated induction of PICyts)

NFκB mediated induction of PICyts has been targeted in various experimental studies [85], [86]. This targeting was performed either by degrading the complex of NFκB or by compromising the resultant gene transcription pathway. The model was evaluated for this intervention by removing the NFκB mediated induction of PICyts as given in Figure 2. To model this intervention, logical parameters given in Table 1 were used with some exceptions given in Table 3. This intervention in non-septic and septic signalling are discussed as CASE 4-N and CASE 4-S, respectively. The models of CASE 4-N and CASE 4-S have been provided as File S9 and File S10, respectively. Simulation of CASE 4-N resulted in a single normal stable state (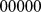) with the absence of PICyts throughout the system (Figure 7). Homeostasis (SCC-6) was seen only between IFN-β and SOCS-1 (Figure S6).

Dynamics of the BRN produced in CASE 4-N and CASE 4-S were comparable except the recurrent induction of TLR4, as discussed above. The results implicate that an immune response could neglect the elevated endotoxemia and allow the pathogen to infect within the immunocompromised host due to the complete absence of PICyts.

#### CASE 5 (intervention in PICyts mediated induction of NFκB and JAK/STAT pathway)

Intervention in PICyts mediated induction of NFκB and JAK/STAT pathway during non-septic signalling is discussed as CASE 5-N whereas in case of sepsis, it is discussed as CASE 5-S. Modelling of CASE 5-N and CASE 5-S were performed using the logical parameters given in Table 1 with exceptions given in Table 3. Moreover, the edge from PICyts towards NFκB-JAK/STAT, as shown in Figure 2, was also removed. The models of CASE 5-N and CASE 5-S have been provided as File S11 and File S12, respectively. The dynamics of both CASES are shown in Figure 8. Events during the simulation of CASE 5-N were slightly different from non-septic signalling (shown in Figure 3), in terms of loss of PICyts mediated induction of JAK/STAT pathway. Moreover, the higher levels of PICyts were not observed throughout the state graph, however, normal levels of PICyts were present. Stable state “
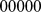
” was present in CASE 5-N, however, in CASE 5-S, this state led towards recurrent induction of TLR4 (trajectories 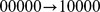, labelled with “η”). Homeostasis due to the cyclic paths SCC-7 and SCC-8 in CASE 5-N were found comparable to SCC-1 and SCC-2 (Figures S7 and Figure S8) except that elevated levels of PICyts were not observed within any cycle.

## Discussion

Methods of high throughput gene expression profiling facilitate the description of complex cellular regulatory networks and present pictures of valuable information about the signalling networks [87], [88]. Regardless of the enormous amount of data associated with molecular and cellular processes produced in various settings, the dynamicity of biological networks in the presence of several interconnected factors still need to be further explored [89]. “Computational systems biology” is a discipline, which is concerned with modelling of experimentally determined values to improve our understanding about BRNs [90]. Computational modelling of BRNs provide useful information about dynamics of various signalling pathways, including control of differentiation process in helper T cells, control of organ differentiation in Arabidopsis thaliana flowers, segmentation during embryogenesis in *Drosophila melanogaster* and TLRs mediated signalling [51]–[53].

Sepsis is a complex pathological state of the body, which involves heterogeneous immune responses of exacerbated inflammation and immunosuppression [82]. Pathophysiology of the sepsis has been associated with pro- or anti-inflammatory responses in different scientific studies, which led to the inconsistency of the overall findings, and failure in its treatment [4]. Some studies associated the deaths in the early phase of sepsis with unrestricted and irrational SIRS in the host [91] and impelled anti-inflammatory treatments [92], [93]. On the other hand, it has also been hypothesized that SIRS is followed by CARS [4], [94], [95]. Moreover, concomitant production of pro- and anti-inflammatory responses have also been demonstrated in polymicrobial infectious challenges, which support the continuous, highly mixed anti-inflammatory response (MARS) and implicated that both pro- and anti- inflammatory cytokines are integral parts of sepsis [96], [97].

TLR4 is a central mediator of LPS induced TH1 or proinflammatory responses, whereas induction of inhibitory mediators can lead the system towards downregulated levels of PICyts [98]. Moreover, binding of cytokines to JAK/STAT receptors induce changes in gene expression levels of various other co-factors necessary for the downregulation of immune response [30]. In order to study the mechanism of sepsis at cellular level, we evaluated the qualitative roles of TLR4 and JAK/STAT signalling with their negative and positive feedback loops necessary to produce effective immune response.

TLR4 and JAK/STAT mediated signalling was designed in the current study by incorporating previous experimental studies associated with interaction of entities and their overall effect in case of sepsis (Figure 1). Reduction of the model was performed to reduce the possible states and trajectories produced during qualitative modelling (Figure 2). In this process, roles of resources in logical parameters were carefully devised so that useful information about the role of any entity present in the model should not be lost. The model was further used with different sets of logical parameters to produce non-septic, septic and intervened signalling to produce dynamics in the form of state graphs. The results of non-septic signalling (Figure 3) were used to compare any interpretations present in this study.

State graphs produced in non-septic signalling were found different from signalling during sepsis in terms of recurring signalling and activation of IFN-β and SOCS-1 in the late phase, which may reflect the immunosuppressive state of the septic patient in the later stages of sepsis. In non-septic signalling, induction of TLR4 and subsequent JAK/STAT signalling mount a successful immune response, which ultimately culminates in downregulated immune response. However, during sepsis, absence of stable state “00000” and recursive signalling through state “00121” can be correlated with the phenomenon of SIRS.

Induction of TLR4 mediated MyD88 and TRIF dependent signalling produced different responses. MyD88 dependent signalling was associated with early induction of PICyts whereas TRIF dependent signalling was associated with late induction of PICyts through Myd88 independent mechanism. This type of early PICyts and delayed IFN-β inductions have been suggested in previous experimental studies associating the time of their onsets in response to pathogen induced immune reaction [99]. Delayed activation from previous experimental studies suggested that IFN-β was produced 24 hours later to *Listeria monocytogenes* infection [100]. Moreover, IFN-β has been observed to attenuate the late hyperinflammatory responses in septic peritonitis [27]. Our study implicates that induction of IFN-β may be present in two stages of septic signalling. In the first stage, IFN-β regulates the levels of PICyts before the induction of SOCS-1, whereas in second stage IFN-β can be induced and downregulate PICyts in late phases of sepsis. Chances for the induction of IFN-β and SOCS-1 were found equal in the late phase of the dynamics of the BRN associated with sepsis and due to this in late phase dynamics of sepsis, there are fewer chances for activation of PICyts to higher levels.

Sequential production and then downregulation of PICyts was observed as one of the interesting phenomenon. The swing in the expression levels of PICyts has already been reported which revealed the pro-inflammation with subsequent immunosuppression [13]. In our study, proinflammatory state can be correlated with those states which had higher activation levels of PICyts (qualitative level “2”) whereas immunosuppression can be correlated with lower levels of PICyts (qualitative level “0 or 1”). SOCS-1 mediated inhibition of PICyts through inactivation of JAK/STAT signalling was found intriguing in the management of immunosuppression. While, inactivation of TLR4 and NFκB mediated induction of PICyts was associated with management of hyperinflammatory responses. This may suggest that therapeutic strategies during the course of sepsis should be devised according to the immune responses and expression levels of SOCS-1 and IFN-β as discussed in other studies for their role in sepsis [31], [47], [82].

*In vitro* studies suggested that recurrent induction of TLR4 through LPS challenge result in the decreased immune response known as LPS tolerance [101]. Our study suggests, that late phase induction of both SOCS-1 and IFN-β may play their roles in LPS tolerance and can produce immunosuppression. Moreover, tolerance may be related with complete absence of PICyts levels even in the continuous signalling through TLR4.

Intervened signalling presented some of the interesting assumptions produced during this study. Mutations or therapeutic intervention of SOCS-1 mediated inhibition of JAK/STAT signalling may result in the non-reversible hyperinflammatory process compared to any other intervention studied here. Moreover, the presence of SOCS-1 can balance the levels of PICyts even during incapable IFN-β mediated inhibition. On the other hand, if non-reversible immunosuppression is required, then intervention of NFκB mediated PICyts expression would produce competitive results.

**Figure 9:**
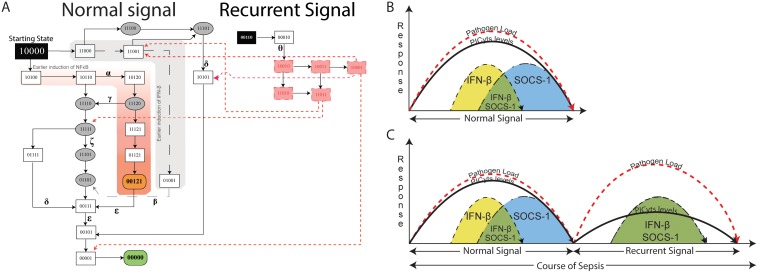
Implication of the study. (A) Edges labelled with Greek small letters and states as nodes are used to represent trajectories associated with different signalling events observed in this study (see legend in Figure 3). Specific states and trajectories of normal and recurrent signalling shown in Figures 3–8 were used to draw the hypothesis shown in (B–C). Possible effects of TLR4 and JAK/STAT signalling on pathogen load, induction pattern of PICyts, IFN-β and SOCS-1 mediated downregulation of PICyts are shown for non-septic (B) and septic (C) cases. During non-septic case, the pattern of IFN-β and then SOCS-1 limits the qualitative levels of PICyts along with the successful reduction of pathogen load. On the other hand, during sepsis, it has been proposed that changed expression pattern of IFN-β and SOCS-1 inhibit the PICyts with resultant increase in the pathogen load.

The overall dynamics of the BRN have been given in Figure 9-A to show the pattern of activation and inactivation of entities. It can be seen that starting from the activation of TLR4, the dynamics actually proceed greatly towards the activation of IFN-β and then SOCS-1 during normal signalling. However, in the case of recurrent signalling, the activation of IFN-β and SOCS-1 at the same time inhibit the activation of PICyts. Based on these predictions, we hypothesize that in normal infections, which do not often lead to sepsis, the phases of regulatory signalling are somewhat different from those seen in case of sepsis as shown in Figure 9 (B–C). This is supported by previous experimental studies in which the continuous presence of pathogens repeatedly induce immune responses and produce oscillatory levels of PICyts [102]. Recurrent infections, which can lead to sepsis, have the capability to induce innate immune responses repeatedly. During this state, PICyts are inhibited to a greater extent which can lead the immune system of the host towards the temporary immunocompromised state. Inhibition may be due to the prior presence of negative regulatory factors such as SOCS-1 and IFN-β in the system, which may be induced in some earlier phase of infection or because of a co-infection. Due to this reason, innate immune response may not efficiently generate an effective PICyts burst to manage the pathogen load. Moreover, the expression pattern of negative regulators such as SOCS-1 and IFN-β can be detrimental in case of sepsis. In normal infections, the pattern of IFN-β and SOCS-1 is sequential, whereas in case of sepsis, this sequential pattern of IFN-β and SOCS-1 may be changed and system becomes more vulnerable towards higher levels of pathogen load due to compromised levels of PICyts.

## Conclusion

In summary, logical modelling of TLR4 and JAK/STAT dependent signalling pathways indicated specifically designed crosstalk mechanism which can induce a successful pathogenic response along with management of hyperinflammation. If entities present in these pathways lose a specific pattern of activation and/or inactivation, then signalling can lead towards diverse outcomes. Using computer-aided qualitative approach, we have tried to highlight these patterns of entities necessary to maintain a balance in a successful immune response. Qualitative results implicated that TLR4 and JAK/STAT pathways induced elevated levels of PICyts with subsequent downregulation. This pattern of activation and then inactivation of PICyts produced homeostasis in the system while changes in the inhibitory role of SOCS-1 created overactive immune responses. The inhibitory role of IFN-β was observed during the initial stages of dynamics, but it is tempting to speculate that SOCS-1 possibly inhibit the role of IFN-β during sepsis but has the ability to manage the hyperinflammatory condition. Overall, this study suggests that intervention in SOCS-1 mediated PICyts inhibition may produce useful results in case of immunocompromised septic patients. On the other hand, intervening the TLR4 or PICyts mediated induction of NFκB-JAK/STAT pathways may be used for the management of hyperinflammatory immune responses. This computational study highlighted many questions with provision of possible answers, which need further experimental investigations. In the future, we will perform *in vitro* experiments to further investigate our predictions and produce explicit insights into the diagnosis and treatment of sepsis by involving IFN-β and SOCS-1.

## Supporting Information

Figure S1 **SCC-1.** This cyclic graph represents the homeostatic regulation of PICyts during physiological signalling dynamics.
(EPS)

Figure S2 **SCC-2.** This cyclic graph represents the homeostasis by SOCS-1 mediated downregulation of PICyts during physiological signalling dynamics.
(EPS)

Figure S3 **SCC-3.** This cyclic graph represents the homeostasis by IFN-β mediated downregulation of PICyts during CASE 3.
(EPS)

Figure S4 **SCC-4.** This cyclic graph represents the homeostasis by SOCS-1 mediated downregulation of PICyts during CASE 3.
(EPS)

Figure S5 **SCC-5.** This cyclic graph represents the homeostasis by SOCS-1 mediated downregulation of PICyts during NFκB downstream signalling in CASE 3.
(EPS)

Figure S6 **SCC-6.** This cyclic graph represents the homeostasis between IFN-β and SOCS-1 during CASE 4.
(EPS)

Figure S7 **SCC-7.** This cyclic graph represents the homeostasis between IFN-β and SOCS-1 during CASE 5.
(EPS)

Figure S8 **SCC-8.** This cyclic graph represents the homeostasis due to SOCS-1 during CASE 5.
(EPS)

Figure S9 **Dummy tendency graphs of TLR4 representing the associated logical parameters used in the modelling of non-septic condition.**
(EPS)

Figure S10 **Dummy tendency graphs of IFN-β representing the associated logical parameters used in the modelling of non-septic condition.**
(EPS)

Figure S11 **Dummy tendency graphs of NFκB representing the associated logical parameters used in the modelling of non-septic condition.**
(EPS)

Figure S12 **Dummy tendency graphs of PICyts representing the associated logical parameters used in the modelling of non-septic condition.**
(EPS)

Figure S13 **Dummy tendency graphs of SOCS-1 representing the associated logical parameters used in the modelling of non-septic condition.**
(EPS)

Table S1 **Other intervention studies.** Other CASES of intervention in signalling were derived by removing specific interactions in Figure 2 along with their logical parameters to observe the possible stable states produced due to each condition.
(DOCX)

File S1 **Model of non-sepsis.xml.**
(XML)

File S2 **Model of sepsis.xml.**
(XML)

File S3 **Model of CASE 1-N.xml.**
(XML)

File S4 **Model of CASE 1-S.xml.**
(XML)

File S5 **Model of CASE 2-N.xml.**
(XML)

File S6 **Model of CASE 2-S.xml.**
(XML)

File S7 **Model of CASE 3-N.xml.**
(XML)

File S8 **Model of CASE 3-S.xml.**
(XML)

File S9 **Model of CASE 4-N.xml.**
(XML)

File S10 **Model of CASE 4-S.xml.**
(XML)

File S11 **Model of CASE 5-N.xml.**
(XML)

File S12 **Model of CASE 5-S.xml.**
(XML)

File S13 **Model of non-sepsis.ginml.**
(GINML)

File S14 **Model of sepsis.ginml.**
(GINML)

File S15 **Model of CASE 1-N.ginml.**
(GINML)

File S16 **Model of CASE 1-S.ginml.**
(GINML)

File S17 **Model of CASE 2-N.ginml.**
(GINML)

File S18 **Model of CASE 2-S.ginml.**
(GINML)

File S19 **Model of CASE 3-N.ginml.**
(GINML)

File S20 **Model of CASE 3-S.ginml.**
(GINML)

File S21 **Model of CASE 4-N.ginml.**
(GINML)

File S22 **Model of CASE 4-S.ginml.**
(GINML)

File S23 **Model of CASE 5-N.ginml.**
(GINML)

File S24 **Model of CASE 5-S.ginml.**
(GINML)

File S25 **Input and output of SMBioNet for the computation of values for logical parameters given in** **Table 1** **.**
(TXT)

File S26 **An informal description of the logical parameters with relevant evidences based on previous experimental studies that forms the basis for selection of a specific value for each logical parameter.**
(DOCX)
