# Supplementary material for: Formal Modelling of Toll like Receptor 4 and JAK/STAT Signalling Pathways: Insight into the Roles of SOCS-1, Interferon-β and Proinflammatory Cytokines in Sepsis
Source: PLoS One. 2014 Sep 25;9(9):e108466. doi: 10.1371/journal.pone.0108466 (PMC4185881; doi:10.1371/journal.pone.0108466)
Supplement: Table S1 — Other intervention studies. Other CASES of intervention in signalling were derived by removing specific interactions in Figure 2 along with their logical parameters to observe the possible stable states produced due to each condition. (DOCX) [file pone.0108466.s014.docx]

**FORMAL MODELLING OF TOLL LIKE RECEPTOR 4 AND JAK/STAT SIGNALLING PATHWAYS: INSIGHT INTO THE ROLES OF SOCS-1, INTERFERON-β AND PROINFLAMMATORY CYTOKINES IN SEPSIS.**

TABLE S1: **Other intervention studies.**  Other CASES of intervention in signalling were derived by removing specific interactions in Figure 2 along with their logical parameters to observe the possible stable states produced due to each condition.

| CASE | Evolving Entity | Target  entity/ies | Removed parameters | Changed parameters | Removed edge/s in Figure 4. | Notes |
| --- | --- | --- | --- | --- | --- | --- |
| 6 | PICyts | NFκB  and  SOCS-1 | $\begin{matrix} K_{NF\kappa B-JAK/STAT}(\{PICyts\})=1 \\ K_{NF\kappa B-JAK/STAT}\left(\{TLR4,\text{ }PICyts\}\right)=1 \\ K_{NF\kappa B-JAK/\mathrm{STAT}}\left(\{IFN-\beta\text{ },\text{ }PICyts\}\right)=1 \\ K_{NF\kappa B-JAK/STAT}\left(\{TLR4,\text{ }PICyts,\text{ }IFN-\beta\}\right)=1 \\ K_{NF\kappa B-JAK/STAT}\left(\{PICyts,\text{ }SOCS-1\}\right)=1 \\ K_{NF\kappa B-JAK/STAT}\left(\{TLR4,\text{ }PICyts,\text{ }SOCS-1\}\right)=1 \\ K_{NF\kappa B-JAK/STAT}\left(\{PICyts,\text{ }IFN-\beta,\text{ }SOCS-1\}\right)=1 \\ K_{NF\kappa B-JAK/STAT}\left(\{SOCS-1,\text{ }TLR4,\text{ }IFN-\beta,\text{ }PICyts\}\right)=1 \\ K_{SOCS-1}\left(\{PICyts,\text{ }IFN-\beta\}\right)=1 \\ K_{SOCS-1}(\{PICyts\})=1 \end{matrix}$ | - | PICyts mediated activation of NFκB-JAK/STAT and SOCS-1 | Stable state “00000” was observed without production of PICyts throughout the system dynamics. |
| 7 | SOCS-1 | IFN-β | $\begin{matrix} K_{IFN-\beta}\left(\{SOCS-1\}\right)=0 \\ K_{IFN-\beta}\left(\{TLR4,\text{ }SOCS-1\}\right)=1 \\ K_{IFN-\beta}\left(\{SOCS-1,\text{ }NF\kappa B\text{ }\}\right)=0 \\ K_{IFN-\beta}\left(\{TLR4,\text{ }SOCS-1,\text{ }NF\kappa B\}\right)=1 \end{matrix}$ | - | SOCS-1 mediated inhibition of IFN-β | Stable state “00000” was observed with production of PICyts during system dynamics. State “00121” was observed during system dynamics. |
| 8 | SOCS-1  and  IFN-β | IFN-β and PICyts | $\begin{matrix} K_{IFN-\beta}\left(\{SOCS-1\}\right)=0 \\ K_{IFN-\beta}\left(\{TLR4,\text{ }SOCS-1\}\right)=1 \\ K_{IFN-\beta}\left(\{SOCS-1,\text{ }NF\kappa B\text{ }\}\right)=0 \\ K_{IFN-\beta}\left(\{TLR4,\text{ }SOCS-1,\text{ }NF\kappa B\}\right)=1 \\ K_{PICyts}(\{SOCS-1\})=0 \\ K_{PICyts}(\{NF\kappa B,\text{ }SOCS-1\})=0 \\ K_{PICyts}(\{NF\kappa B,\text{ }\mathrm{IFN}-\beta,\text{ }SOCS-1\})=0 \\ K_{PICyts}(\{IFN-\beta\})=0 \\ K_{PICyts}(\{IFN-\beta,\text{ }NF\kappa B\})=0 \\ K_{PICyts}\left(\{IFN-\beta,\text{ }SOCS-1\}\right)=0 \\ K_{PICyts}\left(\{NF\kappa B,\text{ }IFN-\beta,\text{ }SOCS-1\}\right)=2 \end{matrix}$ | $K_{PICyts}\left(\{NF\kappa B\}\right)=2$ | SOCS-1 mediated inhibition of IFN-β and PICYts.  IFN-β mediated inhibition of PICyts | Stable states “00000” and “00121” were observed during system dynamics. |
| 9 | TLR4 | NFκB | $\begin{matrix} K_{NF\kappa B-JAK/STAT}\left(\{TLR4\}\right)=1 \\ K_{NF\kappa B-JAK/STAT}\left(\{TLR4,\text{ }IFN-\beta\}\right)=1 \\ K_{NF\kappa B-JAK/STAT}\left(\{TLR4,\text{ }PICyts\}\right)=1 \\ K_{NF\kappa B-JAK/STAT}\left(\{TLR4,\text{ }PICyts,\text{ }IFN-\beta\}\right)=1 \\ K_{NF\kappa B-JAK/STAT}\left(\{TLR4,\text{ }SOCS-1\}\right)=1 \\ K_{NF\kappa B-JAK/STAT}\left(\{TLR4,\text{ }IFN-\beta,\text{ }SOCS-1\}\right)=1 \\ K_{NF\kappa B-JAK/STAT}\left(\{TLR4,\text{ }PICyts,\text{ }SOCS-1\}\right)=1 \\ K_{NF\kappa B-JAK/STAT}\left(\{SOCS-1,\text{ }TLR4,\text{ }IFN-\beta,\text{ }PICyts\}\right)=1 \end{matrix}$ | - | TLR4 mediated induction of NFκB | Stable state “00000” was observed with production of PICyts during system dynamics. State “00121” was observed during system dynamics. |
| 10 | TLR4 | IFN-β | $\begin{matrix} K_{IFN-\beta}\left(\{TLR4\}\right)=0 \\ K_{IFN-\beta}\left(\{TLR4,\text{ }SOCS-1\}\right)=1 \\ K_{IFN-\beta}\left(\{TLR4,\text{ }NF\kappa B\text{ }\}\right)=1 \\ K_{IFN-\beta}\left(\{TLR4,\text{ }SOCS-1,\text{ }NF\kappa B\}\right)=1 \end{matrix}$ | - | TLR4 mediated induction of IFN-β | Stable state “00000” was observed with production of PICyts during system dynamics. State “00121” was observed during system dynamics. |
| 11 | NFκB | TLR4 | $\begin{matrix} K_{TLR4}\left(\{NF\kappa B\}\right)=0 \\ K_{TLR4}\left(\{SOCS-1,\text{ }NF\kappa B\}\right)=0 \end{matrix}$ | - | NFκB mediated inhibition of TLR4 | Stable state “00000” was observed with production of PICyts during system dynamics. State “00121” was observed during system dynamics. |
| 12 | NFκB | IFN-β | $\begin{matrix} K_{IFN-\beta}\left(\{NF\kappa B\text{ }\}\right)=0 \\ K_{IFN-\beta}\left(\{TLR4,\text{ }NF\kappa B\text{ }\}\right)=1 \\ K_{IFN-\beta}\left(\{SOCS-1,\text{ }NF\kappa B\text{ }\}\right)=0 \\ K_{IFN-\beta}\left(\{TLR4,\text{ }SOCS-1,\text{ }NF\kappa B\}\right)=1 \end{matrix}$ | - | NFκB mediated inhibition of IFN-β | Stable state “00000” was observed with production of PICyts during system dynamics. State “00121” was observed during system dynamics. |
