# Supplementary material for: Formal Modelling of Toll like Receptor 4 and JAK/STAT Signalling Pathways: Insight into the Roles of SOCS-1, Interferon-β and Proinflammatory Cytokines in Sepsis
Source: PLoS One. 2014 Sep 25;9(9):e108466. doi: 10.1371/journal.pone.0108466 (PMC4185881; doi:10.1371/journal.pone.0108466)
Supplement: File S26 — An informal description of the logical parameters with relevant evidences based on previous experimental studies that forms the basis for selection of a specific value for each logical parameter. (DOCX) [file pone.0108466.s040.docx]

**Formal Modelling of Toll like Receptor 4 and JAK/STAT Signalling Pathways: Insight into the Roles of SOCS-1, Interferon-β and Proinflammatory Cytokines in Sepsis**

# Description of the logical parameters with relevant evidences.

Below, is the list of entities as shown in Figure 2 with informal description of each logical parameter with relevant evidences based on the previous experimental studies and values given to SMBioNet. The values of some parameters were fixed as single values since they are well-known observations reported in the literature. The output values of SMBioNet have been discussed with their implications in this study. The input and output of SMBioNet can be seen in Supplementary file 25.

# TLR4

1. **Logical Parameter:**

- 1. **Description of the logical parameter:** NFκB and SOCS-1 are active for the TLR4 (see Definition 4 in the methods section for the description of activators or inhibitors as resources for target entity).
  2. **Evidence:** The TLR4 signal is inhibited either due to the absence of an activator entity such as LPS [[1](#_ENREF_1)] or presence of inhibitors such as SOCS-1 and A20. MAL, an adaptor protein in the MyD88 dependent signalling pathway of TLR4 is an important protein (modelled implicitly in the BRN), which is necessary for the activation of NFκB [[2](#_ENREF_2)]. This protein is inhibited by the SOCS-1 [[3](#_ENREF_3),[4](#_ENREF_4)]. Moreover, A20 protein, which has been implicitly modelled in the BRN by an inhibitory edge from NFκB towards TLR4, is transcribed by NFκB, which can in turn form a negative feedback loop to inhibit the NFκB signalling and the ubiquitination process of TRAF6 [[5-8](#_ENREF_5)].
  3. **The values given to SMBioNet as input:** 0 and 1.
  4. **Value given as output by SMBioNet and its implication:** The value “0” was given as output, which implies that TLR4 would inactivate in the presence of SOCS-1 and NFκB.

1. **Logical Parameter:**
   1. **Description of the logical parameter:** SOCS-1 is active and NFκB is inactive for the TLR4**.**
   2. **Evidence:** MAL, an adaptor protein in the MyD88 dependent signalling pathway of TLR4 is an important protein, which is necessary for the activation of NFκB [[2](#_ENREF_2)]. This protein is inhibited by the SOCS-1 [[3](#_ENREF_3),[4](#_ENREF_4)].
   3. **The values given to SMBioNet as input:** 0 and 1.
   4. **Value given as output by SMBioNet and its implication:** The value “0” was given as output, which implies that TLR4 would inactivate in the presence of SOCS-1.
2. **Logical Parameter:**
   1. **Description of the logical parameter:** NFκB is active and SOCS-1 is inactive for the TLR4**.**
   2. **Evidence:** A20 protein has been explicitly modelled in the BRN by an inhibitory edge from NFκB towards TLR4. This represents that as soon as the NFκB activates, it transcribes the A20 along with other genes, which can in turn form a negative feedback loop to inhibit the NFκB signalling and the ubiquitination process of TRAF6 [[5-8](#_ENREF_5)]. TRAF6 has also been modelled in an explicit manner.
   3. **The values given to SMBioNet as input:** 0 and 1.
   4. **Value given as output by SMBioNet and its implication:** The value “0” was given as output, which implies that TLR4 would inactivate in the presence of active NFκB.
3. **Logical Parameter:**
   1. **Description of the logical parameter:** SOCS-1and NFκB are inactive for the TLR4.
   2. **Evidence:** As mentioned in point 2 and 3.
   3. **The values given to SMBioNet as input:** 0 and 1.
   4. **Value given as output by SMBioNet and its implication:** The value “0” was given as output, which represents that TLR4 would inactivate in the absence of active SOCS-1 and NFκB due to the absence of activating factor.

# NFκB and JAK/STAT

1. Logical Parameter:
   1. **Description of the logical parameter:** SOCS-1 is active while activating factors are inactive for the NFκB-JAK/STAT.
   2. **Evidence:** The NFκB or JAK/STAT cannot be activated in the absence of an activator protein or entity [[9](#_ENREF_9),[10](#_ENREF_10)]. Moreover, SOCS-1 has been presented as a negative regulator of NFκB and JAK/STAT pathways in a number of studies [[11-17](#_ENREF_11)].
   3. **The values given to SMBioNet as input:** 0 and 1.
   4. **Value given as output by SMBioNet and its implication:** The value “0” was returned as output, which implies that NFκB-JAK/STAT would inactivate in the absence of activators and in the presence of SOCS-1, which is a negative regulator.
2. Logical Parameter:
   1. **Description of the logical parameter:** TLR4 and SOCS-1 are active for the NFκB-JAK/STAT.
   2. **Evidence:** It has been demonstrated previously that TLR4 is involved in LPS-mediated NFκB activation and subsequent JAK/STAT pathway [[18](#_ENREF_18),[19](#_ENREF_19)]. Moreover, SOCS-1 has been presented as a negative regulator of NFκB and JAK/STAT pathways in a number of studies [[11-17](#_ENREF_11)].
   3. **The values given to SMBioNet as input:** 1 (fixed value).
   4. **Implication:** The value “1” was fixed based on the evidence, which implies that when TLR4 is in an active state then NFκB and JAK/STAT pathway would preferably be in an active state even in the presence of SOCS-1.
3. Logical Parameter:
   1. **Description of the logical parameter:** IFN-β and SOCS-1 are active for the NFκB-JAK/STAT.
   2. **Evidence:** It has been demonstrated previously that along with MyD88 dependent pathway, MyD88 independent pathway through TRIF and TRAF6 (implicitly modelled in BRN through an activation edge from IFN-β towards NFκB) can result in the activation of NFκB [[20](#_ENREF_20),[21](#_ENREF_21)]. Moreover, IFN-β has the ability to activate JAK/STAT pathway [[22](#_ENREF_22)]. On the other hand, SOCS-1 has been presented as a negative regulator of NFκB and JAK/STAT pathways in a number of studies [[11-17](#_ENREF_11)].
   3. **The values given to SMBioNet as input:** 1 (fixed value).
   4. **Implication:** The value “1” was fixed based on the evidence, which implies that when IFN-β is in an active state then NFκB and JAK/STAT pathway would preferably be in an active state even in the presence of SOCS-1.
4. Logical Parameter:
   1. **Description of the logical parameter:** PICyts and SOCS-1 are active for the NFκB-JAK/STAT.
   2. **Evidence:** It has been demonstrated previously that TNF-α, a PICyt, after expression through NFκB can result back in its activation through TNF-α receptors [[23](#_ENREF_23)]. Moreover, PICyts have the ability to activate JAK/STAT pathway [[22](#_ENREF_22)]. On the other hand, SOCS-1 has been presented as a negative regulator of NFκB and JAK/STAT pathways in a number of studies [[11-17](#_ENREF_11)].
   3. **The values given to SMBioNet as input:** 1 (fixed value).
   4. **Implication:** The value “1” was fixed based on the evidence, which implies that when PICyts is in active state, then NFκB and JAK/STAT pathway would preferably also be in an active state even in the presence of SOCS-1.
5. Logical Parameter:
   1. **Description of the logical parameter:** TLR4, IFN-β and SOCS-1 are active for the NFκB-JAK/STAT.
   2. **Evidence:** As mentioned in point number 6 and 7.
   3. **The values given to SMBioNet as input:** 0 and 1.
   4. **Value given as output by SMBioNet and its implication:** The value “1” was returned as output, which implies that when TLR4 or IFN-β would be activated then NFκB and JAK/STAT pathway would also be in an active state even in the presence of SOCS-1.
6. Logical Parameter:
   1. **Description of the logical parameter:** TLR4, PICyts and SOCS-1 are active for the NFκB-JAK/STAT.
   2. **Evidence:** As mentioned in point number 6 and 8. Other references are [[18](#_ENREF_18),[24](#_ENREF_24),[25](#_ENREF_25)].
   3. **The values given to SMBioNet as input:** 0 and 1.
   4. **Value given as output by SMBioNet and its implication:** The value “1” was returned as output, which implies that when TLR4 or PICyts would be in an active state then NFκB and JAK/STAT pathway would also be in an active state even in the presence of SOCS-1.
7. Logical Parameter:
   1. **Description of the logical parameter:** IFN-β, PICyts and SOCS-1 are active for the NFκB-JAK/STAT.
   2. **Evidence:** As mentioned in point number 7 and 8 [[24-27](#_ENREF_24)].
   3. **The values given to SMBioNet as input:** 0 and 1.
   4. **Value given as output by SMBioNet and its implication:** The value “1” was returned as output, which implies that when both IFN-β and PICyts would be in an active state then NFκB and JAK/STAT pathway would also be activated even in the presence of SOCS-1.
8. Logical Parameter:
   1. **Description of the logical parameter:** TLR4, PICyts, IFN-β and SOCS-1 are active for the NFκB-JAK/STAT.
   2. **Evidence:** As mentioned in point number 6, 7 and 8. Other references to previous studies are [[18](#_ENREF_18),[24-27](#_ENREF_24)] .
   3. **The values given to SMBioNet as input:** 0 and 1.
   4. **Value given as output by SMBioNet and its implication:** The value “1” was returned as output, which implies that when TLR4, IFN-β and PICyts would be in an active state then NFκB and JAK/STAT pathway would also be in active state even in the presence of SOCS-1.
9. Logical Parameter:
   1. **Description of the logical parameter:** TLR4 is active and SOCS-1 is inactive for the NFκB-JAK/STAT.
   2. **Evidence:** It has been demonstrated previously that TLR4 is involved in lipopolysaccharide-mediated NFκB activation and subsequent JAK/STAT pathway [[18](#_ENREF_18),[19](#_ENREF_19)].
   3. **The values given to SMBioNet as input:** 0 and 1.
   4. **Value given as output by SMBioNet and its implication:** The value “1” was returned as output, which implies that when TLR4 would activate then NFκB and JAK/STAT pathway would also activate.
10. Logical Parameter:
    1. **Description of the logical parameter:** IFN-β is active and SOCS-1 is inactive for the NFκB-JAK/STAT.
    2. **Evidence:** It has been demonstrated previously that along with MyD88 dependent pathway, MyD88 independent pathway through TRIF and TRAF6 (implicitly modelled in BRN through an activation edge from IFN-β towards NFκB) can result in the activation of NFκB [[20](#_ENREF_20),[21](#_ENREF_21)]. Moreover, IFN-β has the ability to activate JAK/STAT pathway [[22](#_ENREF_22)].
    3. **The values given to SMBioNet as input:** 0 and 1.
    4. **Value given as output by SMBioNet and its implication:** The value “1” was returned as output, which implies that when IFN-β would be active then NFκB and JAK/STAT pathway would also be in an active state.
11. Logical Parameter:
    1. **Description of the logical parameter:** PICyts is active and SOCS-1 is inactive for the NFκB-JAK/STAT.
    2. **Evidence:** It has been analysed previously that TNF-α, a PICyt, after expression through NFκB can result back in its activation through TNF-α receptors [[23](#_ENREF_23)]. Moreover, PICyts have the ability to activate JAK/STAT pathway [[22](#_ENREF_22)]. Other references are [[24](#_ENREF_24),[25](#_ENREF_25),[28](#_ENREF_28)]**.**
    3. **The values given to SMBioNet as input:** 0 and 1.
    4. **Value given as output by SMBioNet and its implication:** The value “1” was returned as output, which implies that when PICyts would be active then NFκB and JAK/STAT pathway would also be in an active state.
12. Logical Parameter:
    1. **Description of the logical parameter:** TLR4 and IFN-β are active, whereas SOCS-1 is inactive for the NFκB-JAK/STAT.
    2. **Evidence:** As mentioned in points 13 and 14.
    3. **The values given to SMBioNet as input:** 0 and 1.
    4. **Value given as output by SMBioNet and its implication:** The value “1” was returned as output, which implies that when TLR4 and IFN-β would be active then NFκB and JAK/STAT pathway would also be in an active state.
13. Logical Parameter:
    1. **Description of the logical parameter:** TLR4 and PICyts are active, whereas SOCS-1 is inactive for the NFκB-JAK/STAT.
    2. **Evidence:** As mentioned in point 13 and 15.
    3. **The values given to SMBioNet as input:** 0 and 1.
    4. **Value given as output by SMBioNet and its implication:** The value “1” was returned as output, which implies that when TLR4 and PICyts would be active then NFκB and JAK/STAT pathway would also be in an active state.
14. Logical Parameter:
    1. **Description of the logical parameter:** PICyts and IFN-β are active, whereas SOCS-1 is inactive for the NFκB-JAK/STAT.
    2. **Evidence:** As mentioned in points 14 and 15**.**
    3. **The values given to SMBioNet as input:** 0 and 1.
    4. **Value given as output by SMBioNet and its implication:** The value “1” was returned as output, which implies that when PICyts and IFN-β would be active then NFκB and JAK/STAT pathway would also be in an active state.
15. Logical Parameter:
    1. **Description of the logical parameter:** Activating factors and SOCS-1 are inactive for the NFκB-JAK/STAT.
    2. **Evidence:** NFκB and JAK/STAT pathways remain inactivated in the absence of activating factors**.**
    3. **The values given to SMBioNet as input:** 0 and 1.
    4. **Value given as output by SMBioNet and its implication:** The value “0” was returned as output, which implies that in the absence of any activator, NFκB and JAK/STAT pathway would be inactive.
16. Logical Parameter:
    1. **Description of the logical parameter:** TLR4, PICyts and IFN-β are active, whereas SOCS-1 is inactive for the NFκB-JAK/STAT.
    2. **Evidence:** As mentioned in points 13-15**.**
    3. **The values given to SMBioNet as input:** 0 and 1.
    4. **Value given as output by SMBioNet and its implication:** The value “1” was returned as output, which implies that in the presence of 3 activators including TLR4, PICyts and IFN-β (MyD88 independent pathway), NFκB and JAK/STAT pathway would be in an active state.

# PICyts

1. Logical Parameter:
   1. **Description of the logical parameter:** SOCS-1 and IFN-β are active for PICyts.
   2. **Evidence:** The suppressor of cytokine signaling-1 (SOCS1) is a potent negative regulator of various cytokines and it has been implicated in the regulation of immune responses, reviewed in [[17](#_ENREF_17)]**.** Moreover, in a previous study it has been observed that IFN-β can shift Th1 immune response to Th2 pattern, thus increasing the production of anti-inflammatory Th2 cytokines, including IL-4, IL-10, and reducing the proinflammatory Th1 cytokines including IFN-γ [[29](#_ENREF_29)]. Other supporting references to previous studies [[30-32](#_ENREF_30)].
   3. **The values given to SMBioNet as input:** 0 and 1.
   4. **Value given as output by SMBioNet and its implication:** The value “0” was returned as output, which implies that in the absence of any activator and presence of SOCS-1 and IFN-β, PICyts would become inactive.
2. Logical Parameter:
   1. **Description of the logical parameter:** NFκB, IFN-β and SOCS-1 are active for PICyts.
   2. **Evidence:** The SOCS-1 is a potent negative regulator of various cytokines and it has been implicated in the regulation of immune responses, reviewed in [[17](#_ENREF_17)]**.** Moreover, in a previous study it has been observed that IFN-β can shift Th1 immune response to Th2 pattern, thus increasing the production of anti-inflammatory Th2 cytokines, including IL-4, IL-10, and reducing the proinflammatory Th1 cytokines including IFN-γ [[29](#_ENREF_29)]. Other supporting references to previous studies [[30-32](#_ENREF_30)].
   3. **The values given to SMBioNet as input:** Range 0-2.
   4. **Value given as output by SMBioNet and its implication:** The value “0” was returned as output, which implies that in the presence of inhibitors such as SOCS-1 and IFN-β, PICyts would become inactivated even in the presence of active NFκB.
3. Logical Parameter:
   1. **Description of the logical parameter:** SOCS-1 is only active, whereas IFN-β is inactive for PICyts.
   2. **Evidence:** As mentioned in point 22.
   3. **The values given to SMBioNet as input:** Range 0-2.
   4. **Value given as output by SMBioNet and its implication:** The value “0” was returned as output, which implies that in the absence of any activator and presence of SOCS-1, PICyts would be inactivated.
4. Logical Parameter:
   1. **Description of the logical parameter:** IFN-β is only active, whereas SOCS-1 is inactive for PICyts.
   2. **Evidence:** As given in point 22.
   3. **The values given to SMBioNet as input:** Range 0-2.
   4. **Value given as output by SMBioNet and its implication:** The value “0” was returned as output, which implies that in the absence of any activator and presence of SOCS-1, PICyts would be inactivated.
5. Logical Parameter:
   1. **Description of the logical parameter:** NFκB and SOCS-1 are active, whereas IFN-β is inactive for PICyts.
   2. **Evidence:** It has been demonstrated that SOCS-1 inhibits cytokine signalling preferably through JAK/STAT pathway [[13](#_ENREF_13),[16](#_ENREF_16),[34](#_ENREF_34)].
   3. **The values given to SMBioNet as input:**  0 (fixed value).
   4. **Implication:** The value “0” was fixed based on the evidence, which implies that the presence of SOCS-1 can result in the inactivation of PICyts meditated signalling even the NFκB is actively involved in the production of PICyts.
6. Logical Parameter:
   1. **Description of the logical parameter:** NFκB and IFN-β are active, whereas SOCS-1 is inactive for PICyts.
   2. **Evidence:** It has been shown that IFN-β can shift the immune response from Th1 to Th2 pattern, and decrease the production of proinflammatory Th1 cytokines such as IFN-γ [[29](#_ENREF_29)] as mentioned in point 22.
   3. **The values given to SMBioNet as input:** 0 (fixed value).
   4. **Implication:** The value “0” was fixed based on the evidence, which implies that the presence of IFN-β can result in the inactivation of PICyts signalling even the NFκB is actively involved in the production of PICyts.
7. Logical Parameter:
   1. **Description of the logical parameter:** IFN-β and SOCS-1 are inactive for PICyts.
   2. **Evidence:** Presence of active NFκB is a necessary event for the production of PICyts [[35](#_ENREF_35)].
   3. **The values given to SMBioNet as input:** Range 0-2.
   4. **Value given as output by SMBioNet and its implication:** The value “0” was returned as output, which implies that in the absence of NFκB, PICyts would become inactive.
8. Logical Parameter:
   1. **Description of the logical parameter:** NFκB is active, whereas IFN-β and SOCS-1 are inactive for PICyts.
   2. **Evidence:** Presence of active NFκB is a necessary event for the production of PICyts [[35](#_ENREF_35)].
   3. **The values given to SMBioNet as input:** 2 (fixed value)
   4. **Implication:** The value “2” was fixed, which implies that NFκB can produce PICyts with an activation level “2”. The possible implication for activation level “2” is to reflect symbolically that PICyts can reach to a greater concentration then any other entity in the system and in turn could activate NFκB and JAK/STAT at threshold level 2 as mentioned in the Methods section.

# SOCS-1

1. Logical Parameter:
   1. **Description of the logical parameter:** No activating factors are present for the SOCS-1.
   2. **Evidence:** Absence of any activator of SOCS-1 would result in its inactivation [[17](#_ENREF_17)].
   3. **The values given to SMBioNet as input:** 0 and 1.
   4. **Value given as output by SMBioNet and its implication:** The value “0” was returned as output, which implies that SOCS-1 would be inactive in the absence of its activators.
2. Logical Parameter:
   1. **Description of the logical parameter:** PICyts are active for the SOCS-1.
   2. **Evidence:** SOCS-1 are induced by several PICyts [[13](#_ENREF_13),[16](#_ENREF_16),[17](#_ENREF_17),[34](#_ENREF_34)].
   3. **The values given to SMBioNet as input:** 1 (fixed value).
   4. **Implication:** The value “1” was fixed based on the evidence, which implies that SOCS-1 would activate in the presence of PICyts.
3. Logical Parameter:
   1. **Description of the logical parameter:** IFN-β is active for the SOCS-1.
   2. **Evidence:** It has been reported that in response to LPS, IFN-β can result in the induction of SOCS-1 [[36](#_ENREF_36),[37](#_ENREF_37)].
   3. **The values given to SMBioNet as input:** 1 (fixed value).
   4. **Implication:** The value “1” was fixed, which implies that SOCS-1 would become active in the presence of IFN-β.
4. Logical Parameter:
   1. **Description of the logical parameter:** PICyts and IFN-β are active for the SOCS-1.
   2. **Evidence:** As mentioned in the point 30 and 31.
   3. **The values given to SMBioNet as input:** 0 and 1.
   4. **Value given as output by SMBioNet and its implication:** The value “1” was fixed, which implies that SOCS-1 would become active in the presence of IFN- β and PICyts.

# IFN-β

1. Logical Parameter:
   1. **Description of the logical parameter:** NFκB and SOCS-1 are active where activating factor is inactive for the IFN-β.
   2. **Evidence:** As TLR4 is involved in the production of IFN-β through TRIF dependent pathway [[38](#_ENREF_38)], so IFN-β cannot be activated in the absence of activated TLR4. Moreover, the inhibitors of IFN-β are SOCS-1 [[39](#_ENREF_39)] and SARM [[40](#_ENREF_40)], implicitly modelled by an edge from NFκB to IFN-β, can inactivate its production or signalling.
   3. **The values given to SMBioNet as input:** 0 and 1.
   4. **Value given as output by SMBioNet and its implication:** The value “0” was returned as output, which implies that IFN- β would be inactivated in the presence of active SOCS-1 and NFκB.
2. Logical Parameter:
   1. **Description of the logical parameter:** TLR4, NFκB and SOCS-1 are active for the IFN-β.
   2. **Evidence:** It has been shown that TLR4 can induce IFN-β, which is MyD88- and PKR (double-stranded RNA-dependent protein kinase)-independent, but TIRAP (Toll-interleukin 1 receptor domain-containing adapter protein)-dependent [[38](#_ENREF_38)] . Moreover, IFN‐β is differentially involved in biological responses through TLR4 [[41](#_ENREF_41)]. Moreover, the inhibitors of IFN-β are SOCS-1 [[39](#_ENREF_39)] and SARM [[40](#_ENREF_40)], implicitly modelled by an edge from NFκB to IFN-β, can inactivate its production or signalling.
   3. **The values given to SMBioNet as input:** 0 (fixed value).
   4. **Implication:** The value “0” was fixed, which implies that in the presence of SOCS-1 and NFκB along with the activated TLR4 signalling, IFN- β would preferably be inactivated.
3. Logical Parameter:
   1. **Description of the logical parameter:** TLR4 and SOCS-1 are inactive whereas NFκB is active for the IFN-β.
   2. **Evidence:** Previous study has reported that SARM [[40](#_ENREF_40)], implicitly modelled in the system by an edge from NFκB to IFN-β, can inactivate production or signalling of TRIF mediated signalling.
   3. **The values given to SMBioNet as input:** 0 and 1.
   4. **Value given as output by SMBioNet and its implication:** The value “0” was returned as output, which implies that active NFκB would result in the inhibition of IFN-β through SARM, implicitly modelled in the BRN.
4. Logical Parameter:
   1. **Description of the logical parameter:** TLR4 and NFκB are inactive whereas SOCS-1 is active for the IFN-β.
   2. **Evidence:** Previous study has shown that SOCS-1 [[39](#_ENREF_39)] can inhibit IFN-β signalling.
   3. **The values given to SMBioNet as input:** 0 and 1.
   4. **Value given as output by SMBioNet and its implication:** The value “0” was returned as output, which implies that active SOCS-1 would result in the inhibition of IFN-β.
5. Logical Parameter:
   1. **Description of the logical parameter:** TLR4 and NFκB are active, whereas SOCS-1 is inactive for the IFN-β.
   2. **Evidence:** It has been shown that TLR4 can induce IFN-β, which is MyD88- and PKR (double-stranded RNA-dependent protein kinase)-independent, but TIRAP (Toll-interleukin 1 receptor domain-containing adapter protein)-dependent [[38](#_ENREF_38)]. Moreover, IFN‐β is differentially involved in biological responses through TLR4 [[41](#_ENREF_41)]. A previous study has reported that SARM [[40](#_ENREF_40)],implicitly modelled in the system by an edge from NFκB to IFN-β, can inactivate its production or signalling.
   3. **The values given to SMBioNet as input:** 0 and 1.
   4. **Value given as output by SMBioNet and its implication:** The value “1” was returned as output, which implies that in the presence of active TLR4, IFN-β could not be inhibited.
6. Logical Parameter:
   1. **Description of the logical parameter:** TLR4 and SOCS-1 are active, whereas NFκB is inactive for the IFN-β.
   2. **Evidence:** In a previous study, analysis of the early TLR4 signalling pathway demonstrated that SOCS1 had no regulatory effect on the activation or on the DNA binding capacity of NFκB. The late effects of LPS are mediated in part through the MyD88-independent pathway activating IRF3 and initiating the production of IFNβ [[28](#_ENREF_28)].
   3. **The values given to SMBioNet as input:** 1 (fixed value).
   4. **Implication:** The value “1” was fixed in speculation that in the presence of active TLR4, the inhibitory effect of SOCS-1 may weakly inhibit IFN-β.
7. Logical Parameter:
   1. **Description of the logical parameter:** TLR4, SOCS-1 and NFκB are inactive for the IFN-β.
   2. **Evidence:** It has been shown that TLR4 can induce IFN-β, which is MyD88- and PKR (double-stranded RNA-dependent protein kinase)-independent, but TIRAP (Toll-interleukin 1 receptor domain-containing adapter protein)-dependent [[38](#_ENREF_38)] . Moreover, IFN‐β is differentially involved in biological responses through TLR4 [[41](#_ENREF_41)].
   3. **The values given to SMBioNet as input:** 0 and 1.
   4. **Value given as output by SMBioNet and its implication:** The value “0” was returned as output, which implies that in the absence of TLR4, IFN- β would become inactive.
8. Logical Parameter:
   1. **Description of the logical parameter:** TLR4 is active, whereas SOCS-1 and NFκB are inactive for the IFN-β.
   2. **Evidence:** It has been shown that TLR4 can induce IFN-β, which is MyD88- and PKR (double-stranded RNA-dependent protein kinase)-independent, but TIRAP (Toll-interleukin 1 receptor domain-containing adapter protein)-dependent [[38](#_ENREF_38)] . Moreover, IFN‐β is differentially involved in biological responses through TLR4 [[41](#_ENREF_41)].
   3. **The values given to SMBioNet as input:** 0 and 1.
   4. **Value given as output by SMBioNet and its implication:** The value “0” was returned as output, which implies that active TLR4 would result in active IFN-β.

# References:

1. Lu Y-C, Yeh W-C, Ohashi PS (2008) LPS/TLR4 signal transduction pathway. Cytokine 42: 145-151.

2. Nagpal K, Plantinga TS, Wong J, Monks BG, Gay NJ, et al. (2009) A TIR domain variant of MyD88 adapter-like (Mal)/TIRAP results in loss of MyD88 binding and reduced TLR2/TLR4 signaling. Journal of Biological Chemistry 284: 25742-25748.

3. Miggin SM (2006) New insights into the regulation of TLR signaling. Journal of Leukocyte Biology 80: 220-226.

4. Mansell A, Smith R, Doyle SL, Gray P, Fenner JE, et al. (2006) Suppressor of cytokine signaling 1 negatively regulates Toll-like receptor signaling by mediating Mal degradation. Nature immunology 7: 148-155.

5. Shembade N, Harhaj EW (2010) A20 inhibition of NFκB and inflammation: targeting E2: E3 ubiquitin enzyme complexes. Cell cycle (Georgetown, Tex) 9: 2481.

6. Shembade N, Ma A, Harhaj EW (2010) Inhibition of NF-κB Signaling by A20 Through Disruption of Ubiquitin Enzyme Complexes. Science 327: 1135-1139.

7. Heyninck K, Beyaert R (1999) The cytokine-inducible zinc finger protein A20 inhibits IL-1-induced NF-[kappa] B activation at the level of TRAF6. FEBS Letters 442: 147-150.

8. Turer EE, Tavares RM, Mortier E, Hitotsumatsu O, Advincula R, et al. (2008) Homeostatic MyD88-dependent signals cause lethal inflamMation in the absence of A20. The Journal of experimental medicine 205: 451-464.

9. Akira S (2000) Toll-like receptors: lessons from knockout mice. Biochemical Society Transactions 28: A488-A488.

10. Adachi O, Kawai T, Takeda K, Matsumoto M, Tsutsui H, et al. (1998) Targeted Disruption of the< i> MyD88</i> Gene Results in Loss of IL-1-and IL-18-Mediated Function. Immunity 9: 143-150.

11. Baetz A, Frey M, Heeg K, Dalpke AH (2004) Suppressor of Cytokine Signaling (SOCS) Proteins Indirectly Regulate Toll-like Receptor Signaling in Innate Immune Cells. Journal of Biological Chemistry 279: 54708-54715.

12. Cooney RN (2002) Suppressors of cytokine signaling (SOCS): inhibitors of the JAK/STAT pathway. Shock 17: 83-90.

13. Fujimoto M, Naka T (2003) Regulation of cytokine signaling by SOCS family molecules. Trends in immunology 24: 659-666.

14. Gingras S, Parganas E, de Pauw A, Ihle JN, Murray PJ (2004) Re-examination of the role of suppressor of cytokine signaling 1 (SOCS1) in the regulation of toll-like receptor signaling. Journal of Biological Chemistry 279: 54702-54707.

15. Johnston JA (2004) Are SOCS suppressors, regulators, and degraders? Journal of Leukocyte Biology 75: 743-748.

16. Yoshimura A (2005) Negative regulation of cytokine and TLR signalings by SOCS and others. Advances in immunology 87: 61.

17. Yoshimura A, Naka T, Kubo M (2007) SOCS proteins, cytokine signalling and immune regulation. Nature Reviews Immunology 7: 454-465.

18. Akira S, Uematsu S, Takeuchi O (2006) Pathogen recognition and innate immunity. Cell 124: 783-801.

19. Chow JC, Young DW, Golenbock DT, Christ WJ, Gusovsky F (1999) Toll-like receptor-4 mediates lipopolysaccharide-induced signal transduction. Journal of Biological Chemistry 274: 10689-10692.

20. Yamamoto M, Sato S, Hemmi H, Hoshino K, Kaisho T, et al. (2003) Role of adaptor TRIF in the MyD88-independent toll-like receptor signaling pathway. Science 301: 640-643.

21. Yamamoto M, Sato S, Hemmi H, Uematsu S, Hoshino K, et al. (2003) TRAM is specifically involved in the Toll-like receptor 4–mediated MyD88-independent signaling pathway. Nature immunology 4: 1144-1150.

22. Schindler C, Levy DE, Decker T (2007) JAK-STAT signaling: from interferons to cytokines. Journal of Biological Chemistry 282: 20059-20063.

23. Barnes PJ (1997) Nuclear factor-κB. The International Journal of Biochemistry & Cell Biology 29: 867-870.

24. Scott MJ, Godshall CJ, Cheadle WG (2002) Jaks, STATs, cytokines, and sepsis. Clinical and diagnostic laboratory immunology 9: 1153-1159.

25. Jia Y, Jing J, Bai Y, Li Z, Liu L, et al. (2011) Amelioration of experimental autoimmune encephalomyelitis by plumbagin through down-regulation of JAK-STAT and NF-κB signaling pathways. PloS one 6: e27006.

26. Fitzgerald KA, Rowe DC, Barnes BJ, Caffrey DR, Visintin A, et al. (2003) LPS-TLR4 Signaling to IRF-3/7 and NF-κB Involves the Toll Adapters TRAM and TRIF. The Journal of experimental medicine 198: 1043-1055.

27. Yamamoto M, Sato S, Hemmi H, Hoshino K, Kaisho T, et al. (2003) Role of adaptor TRIF in the MyD88-independent toll-like receptor signaling pathway. Science's STKE 301: 640.

28. Prêle CM, Woodward EA, Bisley J, Keith-Magee A, Nicholson SE, et al. (2008) SOCS1 regulates the IFN but not NFκB pathway in TLR-stimulated human monocytes and macrophages. The Journal of Immunology 181: 8018-8026.

29. Šega S, Wraber B, Mesec A, Horvat A, Ihan A (2004) IFN-β1a and IFN-β1b have different patterns of influence on cytokines. Clinical neurology and neurosurgery 106: 255-258.

30. Osuchowski MF, Welch K, Siddiqui J, Remick DG (2006) Circulating cytokine/inhibitor profiles reshape the understanding of the SIRS/CARS continuum in sepsis and predict mortality. The Journal of Immunology 177: 1967-1974.

31. Gogos CA, Drosou E, Bassaris HP, Skoutelis A (2000) Pro-versus anti-inflammatory cytokine profile in patients with severe sepsis: a marker for prognosis and future therapeutic options. Journal of Infectious Diseases 181: 176-180.

32. Iskander KN, Osuchowski MF, Stearns-Kurosawa DJ, Kurosawa S, Stepien D, et al. (2013) Sepsis: multiple abnormalities, heterogeneous responses, and evolving understanding. Physiological reviews 93: 1247-1288.

33. Abu-Khabar KS, Armstrong JA, Ho M (1992) Type I interferons (IFN-alpha and-beta) suppress cytotoxin (tumor necrosis factor-alpha and lymphotoxin) production by mitogen-stimulated human peripheral blood mononuclear cell. Journal of Leukocyte Biology 52: 165-172.

34. Palmer DC, Restifo NP (2009) Suppressors of cytokine signaling (SOCS) in T cell differentiation, maturation, and function. Trends in immunology 30: 592-602.

35. Yamamoto Y, Gaynor RB (2001) Role of the NF-kB pathway in the pathogenesis of human disease states. Current molecular medicine 1: 287-296.

36. CRESPO A, FILLA M, RUSSELL S, MURPHY W (2000) Indirect induction of suppressor of cytokine signalling-1 in macrophages stimulated with bacterial lipopolysaccharide: partial role of autocrine/paracrine interferon-α/β. Biochem J 349: 99-104.

37. Dalpke AH, Opper S, Zimmermann S, Heeg K (2001) Suppressors of cytokine signaling (SOCS)-1 and SOCS-3 are induced by CpG-DNA and modulate cytokine responses in APCs. The Journal of Immunology 166: 7082-7089.

38. Toshchakov V, Jones BW, Perera P-Y, Thomas K, Cody MJ, et al. (2002) TLR4, but not TLR2, mediates IFN-[beta]-induced STAT1[alpha]/[beta]-dependent gene expression in macrophages. Nat Immunol 3: 392-398.

39. Song MM, Shuai K (1998) The suppressor of cytokine signaling (SOCS) 1 and SOCS3 but not SOCS2 proteins inhibit interferon-mediated antiviral and antiproliferative activities. Journal of Biological Chemistry 273: 35056-35062.

40. Carty M, Goodbody R, Schröder M, Stack J, Moynagh PN, et al. (2006) The human adaptor SARM negatively regulates adaptor protein TRIF–dependent Toll-like receptor signaling. Nature Immunology 7: 1074-1081.

41. Hoshino K, Kaisho T, Iwabe T, Takeuchi O, Akira S (2002) Differential involvement of IFN‐β in Toll‐like receptor‐stimulated dendritic cell activation. International immunology 14: 1225-1231.
